# Screening of a Thraustochytrid Strain Collection for Carotenoid and Squalene Production Characterized by Cluster Analysis, Comparison of 18S rRNA Gene Sequences, Growth Behavior, and Morphology

**DOI:** 10.3390/md21040204

**Published:** 2023-03-24

**Authors:** Inga K. Koopmann, Bettina A. Müller, Antje Labes

**Affiliations:** ZAiT, Center for Analytics in Technology Transfer of Bio and Food Technology Innovations, Flensburg University of Applied Sciences, 24943 Flensburg, Schleswig-Holstein, Germany

**Keywords:** Stramenopiles, *Thraustochytrium*, *Ulkenia*, *Schizochytrium*, *Oblongichytrium*, Labyrinthulomycetes, chemotaxonomy, microscopy, growth models, cluster analysis

## Abstract

Carotenoids and squalene are important terpenes that are applied in a wide range of products in foods and cosmetics. Thraustochytrids might be used as alternative production organisms to improve production processes, but the taxon is rarely studied. A screening of 62 strains of thraustochytrids *sensu lato* for their potential to produce carotenoids and squalene was performed. A phylogenetic tree was built based on 18S rRNA gene sequences for taxonomic classification, revealing eight different clades of thraustochytrids. Design of experiments (DoE) and growth models identified high amounts of glucose (up to 60 g/L) and yeast extract (up to 15 g/L) as important factors for most of the strains. Squalene and carotenoid production was studied by UHPLC-PDA-MS measurements. Cluster analysis of the carotenoid composition partially mirrored the phylogenetic results, indicating a possible use for chemotaxonomy. Strains in five clades produced carotenoids. Squalene was found in all analyzed strains. Carotenoid and squalene synthesis was dependent on the strain, medium composition and solidity. Strains related to *Thraustochytrium aureum* and *Thraustochytriidae* sp. are promising candidates for carotenoid synthesis. Strains closely related to *Schizochytrium aggregatum* might be suitable for squalene production. *Thraustochytrium striatum* might be a good compromise for the production of both molecule groups.

## 1. Introductions

Carotenoids and squalene belong to the very heterogeneous group of terpenes, sharing the building block isoprene. Carotenoids are tetra- or polyterpenes, comprise over 500 known chemical structures [1] and are produced by a variety of organisms [2,3,4]: mainly plants [5], (micro)algae [6,7,8,9,10], bacteria [11,12,13,14], archaea [15,16], fungi [17,18] and protists [19,20,21]. Many carotenoids offer protective properties against photooxidative stress by quenching reactive oxygen and nitrogen species, photosensitizers, and free radicals [22,23,24,25,26,27]. Antioxidant and anti-inflammatory activities were also observed in vivo [25,28,29,30,31,32], and beneficial effects regarding various health aspects have been described [33,34,35]. This led to the application of carotenoids mainly as food and feed supplements but also as nutraceuticals, pharmaceutics, and in cosmetics [36]. Many carotenoids can be chemically synthesized, but increasing consumer awareness towards sustainable and environmentally friendly products has led to a higher demand for biotechnological production [36]. Many carotenoid-producing organisms do not synthesize large amounts of these substances or are not suitable for high-density cultivation. Thus, biotechnological production processes often suffer from low yields.

Squalene is a triterpene that is ubiquitous in higher organisms. It is abundant in human skin surface lipids [37] and an intermediate in sterol biosynthesis in plants and animals [38,39]. It is mainly used in cosmetics as an antioxidant and hydrating agent [40,41] but also in pharmaceuticals, e.g., as an adjuvant in vaccines [42,43,44], in nutraceuticals, and food products [42]. It is highly abundant in the liver of certain shark species [45,46,47,48,49], from which it was also first isolated [50]. It can also be extracted from various fruits, legumes, grains, seeds, and nuts, especially from olives and amaranth seeds [51,52,53,54,55]. Although plant-based resources have a growing market share, some squalene is still derived from endangered sharks, which has been heavily criticized. As the market size value of squalene is expected to continue to grow in the coming years [41,42], a sustainable and easily scalable resource for squalene must be found.

Different optimization approaches are necessary to improve the efficiency of carotenoid and squalene production. The selection of an optimal production organism is one of them. Thraustochytrids is a promising group of organisms, being a family of marine unicellular organisms. They are eukaryotic, saprobic protists and occur ubiquitously in the marine ecosystem. Thraustochytrids play an important part in the microbial loop as nutrient recyclers and act as partners in various relationships with algae and other marine organisms [56,57]. The taxonomy of the thraustochytrids still needs clarification. Since their first description in 1934 [58], their classification has changed, from belonging to fungi to oomycetes [59] and finally to a phylogenetic group of its own together with the labyrinthulids in the polyphyletic kingdom of Protista [60]. 18S rRNA gene comparison confirmed their independency from oomycetes, and they were associated with the stramenopiles [61,62]. On a lower taxonomic level, the assignment of various strains to different orders and genera has been repeatedly revisited, still in recent years [62,63,64,65,66,67,68].

Some strains are successfully used for the production of long-chain *ω*-3-fatty acids, especially docosahexaenoic acid (DHA) (C22:6), at laboratory [69,70,71,72,73,74,75] and industrial scale [76]. Thraustochytrids are further known for the synthesis of squalene [69,70,71,77,78,79] and carotenoids, particularly astaxanthin, *β*-carotene, canthaxanthin, echinenone, and phoenicoxanthin [19,20,21,63,64,74,80,81,82,83]. The heterotrophic cultivation is a major advantage over microalgae, the main carotenoid-synthesizing organisms in large-scale biotechnological production. Microalgae have to be grown phototrophically in most cases, which means high operational effort and costs. Thraustochytrids have a great potential to reach high cell densities of up to 170 g/L [84,85] and can be cultivated in fermentation processes using various waste streams [69,70,71,86]. Therefore, an optimization of the production rate of carotenoids and squalene in comparison to established processes is very likely. Nevertheless, there is still a lack of knowledge regarding this very heterogeneous group of organisms. The fact that today’s production strains are mainly members of the genus *Schizochytrium* shows that the diversity of this group is not fully used [76].

A few production strains and those commonly used in the laboratory are fairly well described. Still, for the vast diversity of thraustochytrids, only few coherent datasets combine taxonomic data with optimal cultivation parameters, morphology, and secondary metabolite patterns [63,64,87]. Such combined datasets have been used and proposed to improve the understanding and quality of taxonomic arrangements [63,64] and provide better access to industrial applicability. A major problem in this specific group is the high variability in morphology and metabolite patterns, partly also depending on different cultivation parameters.

This work aims to present a better and more coherent picture of this group by a multilevel screening of a strain collection of marine thraustochytrids. We used design of experiment (DoE) and modeling tools to explain the growth of various strains. 18S rRNA gene-based phylogeny was correlated with the production of various carotenoids and squalene, and microscopic morphology. The objective was to show and describe that strains similar at molecular level have similar growth characteristics, morphology, and metabolite patterns with a specific focus on squalene and carotenoids. This picture shall enable and simplify the classification and cultivation of new and not studied strains, not least to test their applicability on an industrial scale for producing valuable substances.

## 2. Results

### 2.1. Molecular Identification

**Figure 1:**
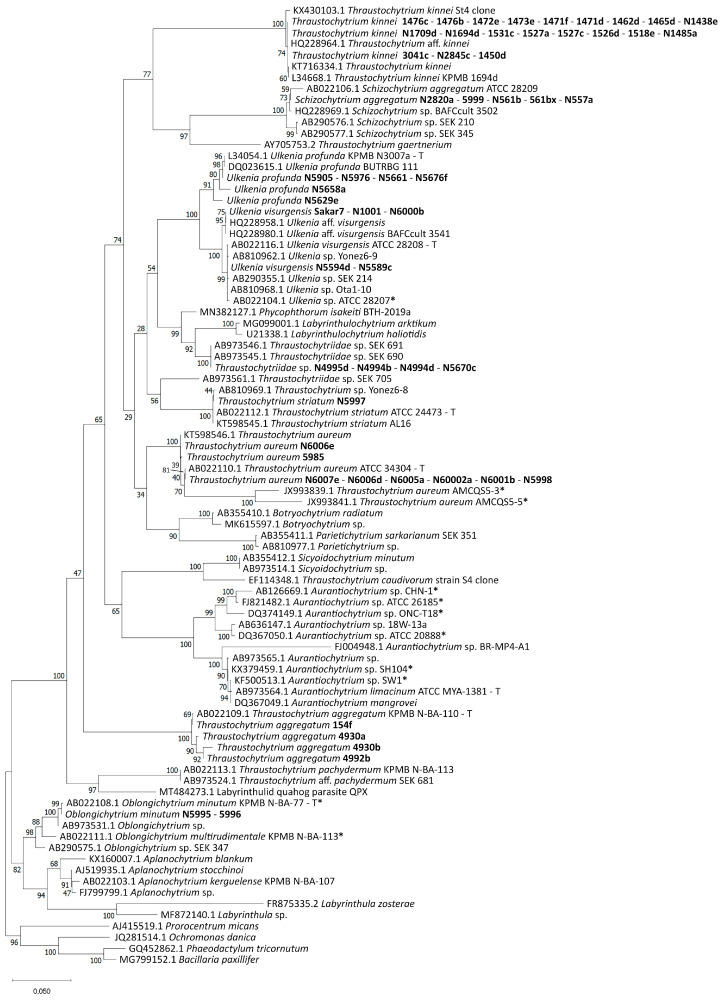
Phylogenetic tree based on 18S rRNA genes created using the maximum-likelihood method and the Tamura–Nei nucleotide substitution model. The final dataset contained 1378 base positions. Bootstrap values are shown for 1000 replicates. Type strains are marked with a “T”. Strains that were reclassified in this study are marked with an asterisk. Strains from the mFSC collection are highlighted bold. Accession numbers, names of taxa and strain labels are shown for the sequences retrieved from GenBank. Strains with no calculated evolutionary distance are on the same branch.

The 18S rRNA sequences were compared with those in the GenBank database using the Basic Local Alignment Search Tool (BLAST). The majority of strains of the collection were assigned to already described genera of the Thraustochytriaceae, namely *Thraustochytrium* spp., *Ulkenia* spp., *Thraustochytriidae* sp., *Schizochytrium aggregatum*, and *Oblongichytrium minutum* (Table S1). Only one group of strains (N6421, N6422, N6423, N6424, N6523) was closer related to *Paranamyces uniporus* (NCBI accession number MT731025.1), which belongs to the Rhizophydiales. This group was not used for the final alignment and tree formation. The phylogenetic tree (Figure 1) contained two main clades and the outgroup. One contained members of the Thraustochytriaceae and most of the strains from the collection. The other one contained members of the Thraustochytriaceae and Labyrinthulaceae. Only two strains from the collection (N5995 and 5996) were located here, being closely related to *Oblongichytrium minutum* (AB022108.1). Within the branches of the two clades, many of the strains of the collection had little or no evolutionary distance. Twenty of the strains were closely related to *Thraustochytrium kinnei* with only small or no evolutionary distance to each other. For strain N1694d, the sequence was already available (*T. kinnei* L34668.1) [61]. The 18S rRNA gene sequence of this strain (here with number N1694d) was similar but not identical. Amplification of 18S rRNA genes of most strains related to *S. aggregatum* was not possible with primers T18S1F and T18S5R. Only shorter segments were amplified.

### 2.2. Growth Studies

Design of experiment, model estimation, and selection were performed to find optimal cultivation conditions for the thraustochytrids and to compare their growth behavior. Three models (Equations (1)–(3) were estimated and compared to find the best description for growth at a constant salt concentration (15 g/L): In the simplest model (Equation (1)/model 1), the quadratic influence of all parameters was considered. Model 2 (Equation (2)) reduced the quadratic influences to those of glucose and yeast extract and included the interaction term of yeast extract and glucose. Model 3 (Equation (3)) included further interaction terms.

In total, the experiment was performed with 30 strains. Some strains did not grow at all (*T. kinnei* 1438e, *U. profunda* N5976, N5629e) or not sufficiently for model estimation (*U. profunda* N5905). These strains were excluded from the model evaluation. The models of the remaining 26 strains were summarized and compared by the median of the adjusted coefficients of determination. The highest values were reached by model 3, followed by model 2 and model 1, with a median of the adjusted R² of 0.904, 0.775, and 0.689, respectively. Comparing the adjusted coefficients of determination directly, the third model (Equation (3)) scored a higher adjusted R² for 65% of the strains. The best-describing model varied for some of the clusters detected in the phylogenetic tree. *U. visurgensis* N6000b and Sakar 7 were best described by model 1. *U. visurgensis* N5589c, N5594d, and *U. profunda* N5658a were best described by model 2. *Thraustochytriidae* sp. N4994d, N4995d, and N5670c, *T. kinnei* N1709d, N1694d, 14766c, 1465d, 1462d, and 3041c, and *T. aggregatum* 4992b and 154f were best described by model 3. The coefficients of determination and *p*-values of all models can be found in the supplement (Table S2). Model 1 showed that the quadratic influence of pH or phosphate had no significant influence on the growth, except for one strain, and was not further evaluated. Due to the reduction of the degrees of freedom by the addition of interaction terms, model 3 did not display many parameters as significant. Thus, for a general impression of the importance of the parameters and better clarity and comparability, model 2 was chosen to carefully compare all strains with an adjusted R² ≥ 0.7 and a significant *p*-value (σ = 0.05). These were *U. visurgensis* Sakar7, N6000b, N5589c, N5594d, *Thraustochytriidae* sp. N4994d, N4995d, N5670c, *T. aureum* N6007e, N6006d, *T. kinnei* N1709d, N1694d, N1476c, 1462d, *T. aggregatum* 4992b, and 154f.

Glucose and yeast extract addition had a generally positive influence on growth. The linear parameter for yeast extract concentration was considered significant (σ = 0.05 or σ = 0.01) for 93% of the tested strains, followed by glucose with a significant linear influence on the growth of 73% of the named strains. The quadratic coefficients of both (glucose and yeast extract) were most often slightly negative but insignificant. The parameters for the interaction of glucose and yeast extract were primarily small but positive. It was considered significant for the growth of 27% of the strains. This resulted in predicted high optimal concentrations of up to the maximum of glucose (60 g/L) and yeast extract (15 g/L) for most strains (Table 1 and Table S3). *U. visurgensis* Sakar 7 and N6000b showed lower optimized glucose concentrations of 46 and 32 g/L, respectively. Strain N6000b also had a lower optimized yeast extract value of 10.1 g/L. Likewise, the optimization for the results of *Thraustochytriidae* sp. N4995d and *T. aggregatum* 154f indicated that lower glucose and yeast extract concentrations were optimal. The influence of the pH value and the addition of phosphate was considered significant only in under 15% of the strains. The coefficient of the pH value was negative for most strains, whereas the coefficient for the addition of phosphate was balanced positive and negative. Thus, the optimized pH was the minimum of 6.5 for all strains except for *T. aggregatum* 154f. The optimized additional phosphate concentration was either 0 or 0.5 g/L.

Many of the strains that were clearly better described by model 3 (*S. aggregatum* N2820a, 5999, 561bx, *T. aureum* N5998, 5986, *O. minutum* 5996, and *T. striatum* N5997) still showed a growth optimum at 50-60 g/L glucose, but some had a lower optimum for yeast extract (*T. aureum* N5998, 5986, and *O. minutum* 5996). Only *T. kinnei* 1465d, 3041c, and *O. minutum* N5995 had a growth maximum at lower glucose (0 und 26 g/L) and varying yeast extract concentration (15 and 0.5 g/L).

*T. aureum* N6006d, N6007e, 5985, *T. aggregatum* 4992b, 154f, and *T. striatum* N5997 yielded the highest biomasses of all strains. Their average yield varied between 4 and 7 g/L. All these strains grew best on DoE medium 8 or 9 (Table S4). The highest yield of 27 g/L was obtained from N6007e on medium 8, regardless of the salt concentration. *Thraustochytriidae* sp. N4995d and *S. aggregatum* 561bx, and 5999 yielded just over 2 g/L on average. Most of the other strains yielded less than 2 g/L.

Sixteen of the analyzed strains were additionally cultivated on media with a higher salinity. Model 2 was extended by the linear influence of salt concentration. A slightly but significant negative influence (σ = 0.05) was found in *Thraustochytriidae* sp. N4995d. A significant positive influence (σ = 0.01) was observed in *O. minutum* N5995, *U. visurgensis* Sakar7, and *U. profunda* N5905 and N5629e. The effect was particularly clear for the latter two. These strains only grew on one of the 15 media with a lower salt concentration (DoE 13 and 3, respectively) and on most of the media with a higher salt concentration of 30 g/L. Of the media with the higher salt concentration, they yielded the highest biomass on media 13 and 6, respectively.

### 2.3. Target Molecules

The occurrence of various carotenoids and squalene was analyzed in the strains. The following carotenoids were detected by comparison with standards: astaxanthin, astaxanthin monopalmitate, phoenicoxanthin, canthaxanthin, 9*Z*-canthaxanthin, echinenone, lycopene, and *β*-carotene. Neither lutein, zeaxanthin, antheraxanthin, nor rhodoxanthin were observed in comparison to the standards. Diastereomers of astaxanthin were also detected. Carotenoids were found in all tested strains except for those closely related to *T. kinnei* (1462d, 1465d, N1476c, N1694d, N1709d), *T. aggregatum* (154f, N4992b), and *O. minutum* (N5995). Squalene was detected in all strains.

### 2.4. Cluster Analysis of Carotenoid Composition

**Figure 2:**
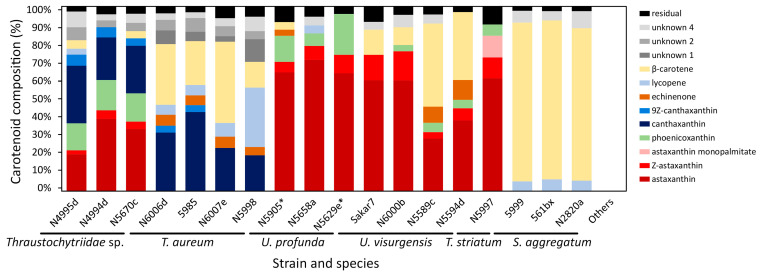
Carotenoid composition of the analyzed strains. Mean values of the individual experiments from each strain whose share in the total carotenoids exceeded 3% are displayed individually (Table S5). Diastereomers of astaxanthin are summarized as “*Z*-astaxanthin”. “Others” comprises all tested strains assigned to *T. kinnei* (N1694d, 1462d, 1465d, N1476c, N1709d, 3041c), *T. aggregatum* (4992b, 154f), and*O. minutum* (N5995). Strains that grew only on media with high salinity are marked with asterisks.

**Figure 3:**
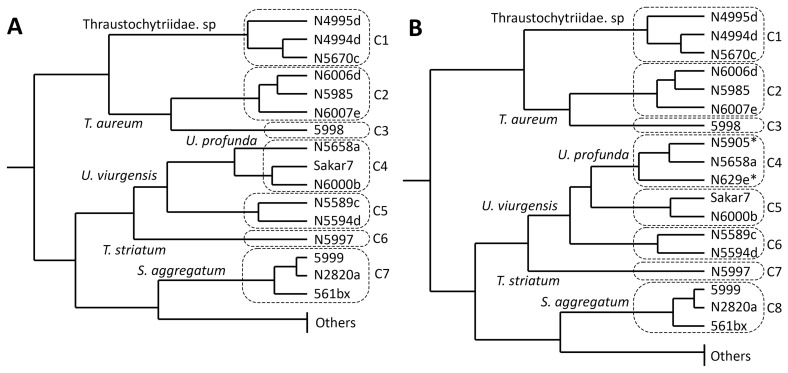
Hierarchical clustering of the analyzed strains based on their carotenoid profiles. Clusters calculated by k-means algorithm were highlighted by dashed boxes. (**A**) Clusters excluding carotenoid patterns of *U. profunda* N5629e and N5905. (**B**) Clusters including carotenoid patterns of *U. profunda* N5629e and N5905 (marked with asterisks) cultivated on media with a high salt concentration. “Others” comprises all tested strains assigned to *T. kinnei* (N1694d, 1462d, 1465d, N1476c, N1709d, 3041c), *T. aggregatum* (4992b, 154f), *and O. minutum* (N5995).

A comparison of the carotenoid composition (Figure 2, Table S5 and S6) by k-means and hierarchical cluster analysis (Figure 3) indicated eight different clusters of strains.

The first cluster was characterized by a balanced ratio of astaxanthin and canthaxanthin (cluster means of 30% and 28%, respectively) and some phoenicoxanthin (cluster mean of 16%). It contained all strains closely related to *Thraustochytriidae* sp. The second cluster comprised three of the four strains closely related to *T. aureum*, characterized by major proportions of canthaxanthin (cluster mean of 32%) and *β*-carotene (cluster mean of 35%). The fourth strain of this group (*T. aureum* N5998) was, from a phylogenetic view, closely related to two of the strains from that cluster. It was only associated with that cluster because of still comparably high canthaxanthin (cluster mean of 18%) and *β*-carotene values (cluster mean of 14%). However, it was not included, mainly because of its outstanding lycopene proportion. It was the highest of all strains at 34%. The fourth and fifth cluster contained all strains closely related to *Ulkenia* species. The fourth clustered *U. profunda* N5658a and *U. visurgensis* Sakar7 and 6000d and displayed predominantly a high astaxanthin content (cluster mean of 64%). The fifth was characterized by astaxanthin (cluster mean of 33%) and *β*-carotene (cluster mean of 42%) and contained *U. visurgensis* N5589c and N5594d. *T. striatum* N5997 had a separate position in the tree but was located in close proximity to the *Ulkenia* clusters. It had a similar astaxanthin proportion (cluster mean of 62%), but produced also an astaxanthin ester, which could not be found in any of the other strains. The last comparable cluster comprised strains closely related to *S. aggregatum* with a high proportion of *β*-carotene (cluster mean of 88%). Strains closely related to *T. kinnei*, *T. aggregatum*, and *O. minutum* were clustered because no carotenoids were detected.

### 2.5. Cluster Analysis of Carotenoid Composition, including Strains with a High Salt Affinity

Comparison of carotenoid profiles emerging on media with high (30 g/L) and low (15 g/L) salt concentration showed only small differences between the same strain (Table S7 and Figure S1). *U. profunda* N5629e and N5905 did produce almost no biomass on media with a salt concentration of 15 g/L. Therefore, their carotenoid patterns were evaluated only by the biomass grown on the media with the higher salinity. They were similar to those of *U. profunda* N5658a (Figure 2). Inclusion in cluster analysis resulted in the division of cluster 4 (Figure 3). All strains related to *U. profunda* (N5658a, N5629e, N5905) now clustered with a higher mean of astaxanthin (67%) and some phoenicoxanthin (cluster mean of 15%). *U. visurgensis* N6000b and Sakar7 formed a separate cluster with mainly astaxanthin (cluster mean of 60%) and *β*-carotene (cluster mean of 12%). *U. visurgensis* N5594d and N5589c remained unaffected.

### 2.6. Target Molecule Content and Yield in the DoE Studies

**Figure 4:**
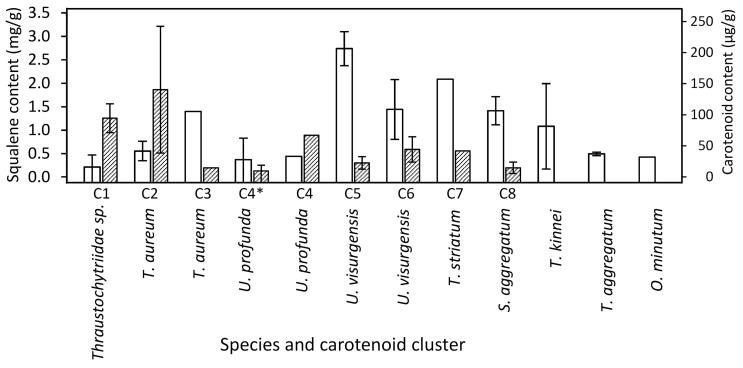
Mean squalene (empty bars) and carotenoid (shaded bars) content of strains belonging to the same carotenoid cluster (Figure 3B). Strains that grew only on media with high salinity are marked with asterisks. Whiskers indicate the standard deviation between the different strains of a cluster. Dry weight was approximated using data from the other trials.

Mean total carotenoid content varied between 0 and 246 µg/g (maximum in *T. aureum* 5985). Clusters 1 and 2 comprised the strains with the highest carotenoid content, whereas squalene was generally more abundant in *T. striatum* N5997 and some *Ulkenia* strains (Figure 4). It has to be noted that these values are means over all the various media used in the DoE. Strains performed better on some of the individual media. e.g., *Thraustochytriidae* sp. N4994d, *T. aureum* N6006d, N6007e, and *U. visurgensis* Sakar7, N5589c showed a higher carotenoid content on medium 8 than in the mix or on the other individually tested media. *T. aureum* 5985 possessed the highest total carotenoid content of 307 µg/g on medium 9. Most strains related to *Thraustochytriidae* sp. and *T. aureum* also produced high biomass. Thus, the carotenoid yield was also high and resulted in 6 mg/L for *T. aureum* 5985 on medium 9.

The mean squalene content varied between 0.02 mg/g (*Thraustochytriidae* sp. N4995d) and 3 mg/g (*U. visurgensis* N6000b). The highest squalene contents observed were 13 mg/g and 12 mg/g in *T. striatum* N5997 on medium 11, and in *O. minutum* N5995 on medium 14 with a high salt concentration, respectively.

### 2.7. Regression Analysis of Target Molecules

For a more detailed insight into the carotenoid and squalene synthesis based on the dependency of the media composition, target molecules in *T. striatum* N5997 were analyzed on all media from the DoE except for number 8, on which it did not grow. In addition, the growth regression was repeated to find a model that described growth better than the previously obtained models (model 1-3). The resulting regression had an adjusted R² of 0.8819. Due to few degrees of freedom, only the optimized parameters were evaluated, but not their individual influences. Model optimization indicated that the maximum glucose concentration (60 g/L) and a small yeast extract concentration (3.9 g/L) maximized the biomass yield (Table 2). Optimization of squalene content resulted in a minimum glucose concentration and maximum yeast extract concentration. An intermediate glucose and yeast extract concentration was considered advantageous for squalene yield.

Carotenoids found in *T. striatum* N5997 were mainly astaxanthin, its diastereomers, and an ester (85.4% of the carotenoids in total). Phoenicoxanthin (6.3%), lycopene (2.6%), *β*-carotene (1.5%), canthaxanthin and its diastereomer (1.2%), and two unknown carotenoids (2.9%) were observed to a smaller extent. Carotenoid composition changed slightly depending on the medium composition (Table S8 and Figure S2). In contrast to squalene, a high glucose (48–60 g/L) but minimal yeast extract concentration (0.5 g/L) was found to be beneficial in optimizing total and individual carotenoid content. The highest absolute carotenoid yield was predicted at maximum glucose and minimum yeast extract concentration.

### 2.8. Effect of the Medium Composition on the Target Molecules

A selection of strains was used to replicate the experiment on media 6, 9, and 15. Medium 9 was chosen because the highest biomass for most of the strains was yielded here. Medium 15 was most similar to the optimized parameters for a high carotenoid content. Medium 6 was chosen for its reduced glucose and medium yeast extract content, which were thought to increase squalene content while promoting growth, as suggested by the optimized media in the regression of *T. striatum* N5997.

Cell dry weight (CDW) was determined in those strains that yielded the highest biomasses on medium 9 (N6007e, 5985, N6006d, 5996, N2820a, 5999). The mean CDW was 34.8 ± 6.1% *w/w*. The highest amount of extract was obtained from *T. aureum* N6006d and 5985 and from *S. aggregatum* 5999 and N2820a on media 9 and 15 (Table S9). These extracts had a fatty appearance.

**Figure 5:**
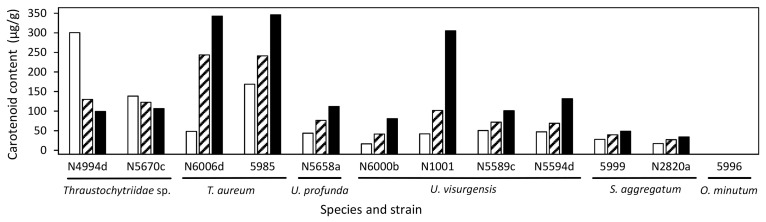
Carotenoid content in selected strains grown on DoE media 6 (empty bars), 9 (shaded bars), and 15 (filled bars).

**Figure 6:**
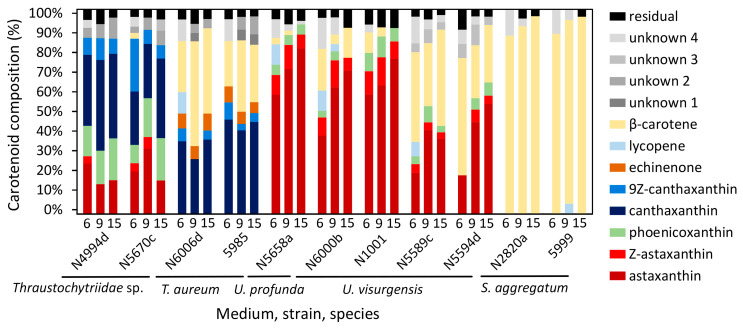
Carotenoid composition of the analyzed strains cultivated on medium 6, 9, and 15. Individually displayed are only carotenoids whose contribution to the total carotenoids was above 3% (Table S10). Diastereomers of astaxanthin are summarized as “*Z*-astaxanthin”.

Carotenoid content varied between 0 and 346.1 µg/g in the different strains and media (Figure 5). For all strains except *Thraustochytriidae* sp. N5670c and N4994d, the highest carotenoid content was measured when grown on medium 15, with maximum values of 343 and 346 µg/g in *T. aureum* N6006d and 5985, respectively. The carotenoid compositions were consistent with those described in the cluster analysis. The media nevertheless had an influence on the exact carotenoid composition (Figure 6 and Table S10).

**Figure 7:**
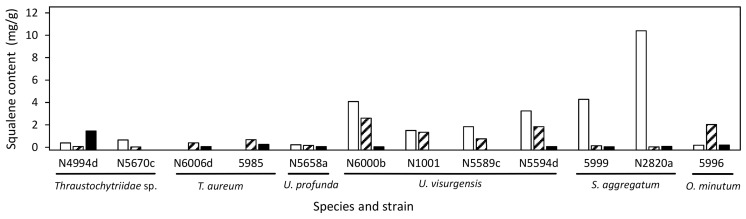
Squalene content in selected strains grown on DoE medium 6 (empty bars), 9 (shaded bars), and 15 (filled bars).

The highest squalene content was most often reached on medium 6 (Figure 7). *S. aggregatum* N2820a and 5999 showed especially high squalene content on medium 6 but a generally low carotenoid content. The highest squalene content was 10.4 mg/g in *S. aggregatum* N2820a. *T. aureum* N6006d and 5985 as well as *Thraustochytriidae* sp. N5670c and N4994d showed the opposite pattern with generally high carotenoid but low squalene contents.

### 2.9. Comparison of Biomass Yield and Target Molecule Content in Cultures Cultivated in Liquid and on Solid Medium

**Figure 8:**
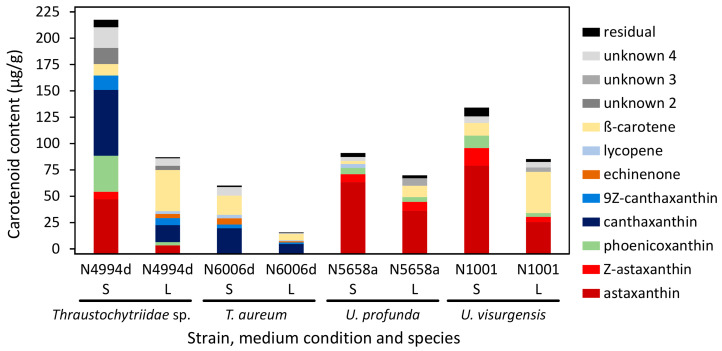
Carotenoid content and composition of four different strains cultivated on DoE medium 7 either in liquid (L) culture or on solid medium (S). Individually displayed are only carotenoids whose contribution to the total carotenoids was above 3% (Table S11). Diastereomers of astaxanthin are summarized as “Z-astaxanthin”.

Representative strains of four species with a high carotenoid production were chosen for a comparative study. *Thraustochytriidae* sp. N4994d, *T. aureum* N6006d, *U. profunda* N5658a, and *U. visurgensis* N1001 were cultivated on DoE medium 7 either in liquid or solid culture. In liquid culture, biomass densities of 1.1, 1.0, 0.6, and 0.7 g/L were reached for N4994d, N6006d, N5658a, and N1001, respectively. On solid medium, the CDW of the cultures scraped from the agar was 25.0, 30.7, 29.7, and 28.1% *w/w*, and biomass yields of 1.4, 1.8, 1.5, and 1.3 g/L were harvested for strains N4994d, N6006d, N5658a, and N1001, respectively. Extract yield was higher on the solid media (Table S9). The carotenoid content varied from 16 µg/g in N6006d in liquid medium to 218 µg/g in N4994d on solid medium (Figure 8). The carotenoid compositions of the strains cultivated on solid medium were similar to those observed in the previous experiment. *U. visurgensis* N1001 fitted to the other strains of the genus *Ulkenia*. A higher carotenoid content was measured in all strains cultivated on solid medium than in liquid medium. It was 1.3-fold higher in *U. profunda* N5658a and 3.8-fold higher in *T. aureum* N6006d. The carotenoid patterns changed according to the cultivation condition (Figure 8 and Table S11). The percentage of *β*-carotene increased in all strains when cultivated in liquid medium, whereas the percentage of astaxanthin, phoenicoxanthin, and canthaxanthin decreased. *Thraustochytriidae* sp. N4994d exhibited the greatest differences in carotenoid composition when the cultivation medium was changed.

Squalene content was similar in all strains except for N1001. For N4994d, N6006d, and N5658a, it varied between 0.3 and 0.7 mg/g when cultivated on solid medium and between 2.8 and 2.9 mg/g when cultivated in liquid medium. *U. visurgensis* N1001 showed higher contents of 6.0 and 8.6 mg/g when cultivated on solid and in liquid medium, respectively. Squalene was between 1.4 and 9.0 times higher when the cultivation was performed in liquid medium.

### 2.10. Unknown Metabolites

Besides those carotenoids that were identified by the comparison with standards, six other substances might be classified as carotenoids. Four of them exceeded the threshold of 3% applied for the cluster analysis and carotenoid patterns.

Unknown 1 was only present in all species belonging to *T. aureum*. Its retention time was very similar to that of phoenicoxanthin (9.0 min) but had a different absorption spectrum with a single maximum at 448 nm (Table 3). Unknowns 2 and 3 were present in various species. Retention times were at 10.4 and 14.8 min. They had similar UV/Vis absorption maxima as echinenone (461 nm and 462 nm, retention time at 16.5 min). The mass spectrum of unknown 2 had two main peaks at *m*/*z* 566.4 and *m*/*z* 549.8. Unknown 4 was present in *T. aureum*, *Thraustochytriidae* sp., *Ulkenia* spp., and *S. aggregatum*. Its retention time was 21.9 min, and it was close to that of astaxanthin monopalmitate and *β*-carotene. It had a characteristic UV/Vis absorption spectrum with two peaks and a shoulder and maxima at 461 nm and 489 nm.

One group of substances was present in *O. minutum* N5995. It was only observed on DoE medium 14 with a high salt concentration. The substances had a very characteristic UV/Vis absorption spectrum. These were porphyrins rather than carotenoids. The substance with the highest peak (retention time at 6.8 min) had one large maximum at 401 nm and further small maxima at 504, 538, 574, and 628 nm. The main observed mass was *m*/*z* 563.4, accompanied by further substances with similar absorption spectra.

### 2.11. Morphology

**Figure 9:**
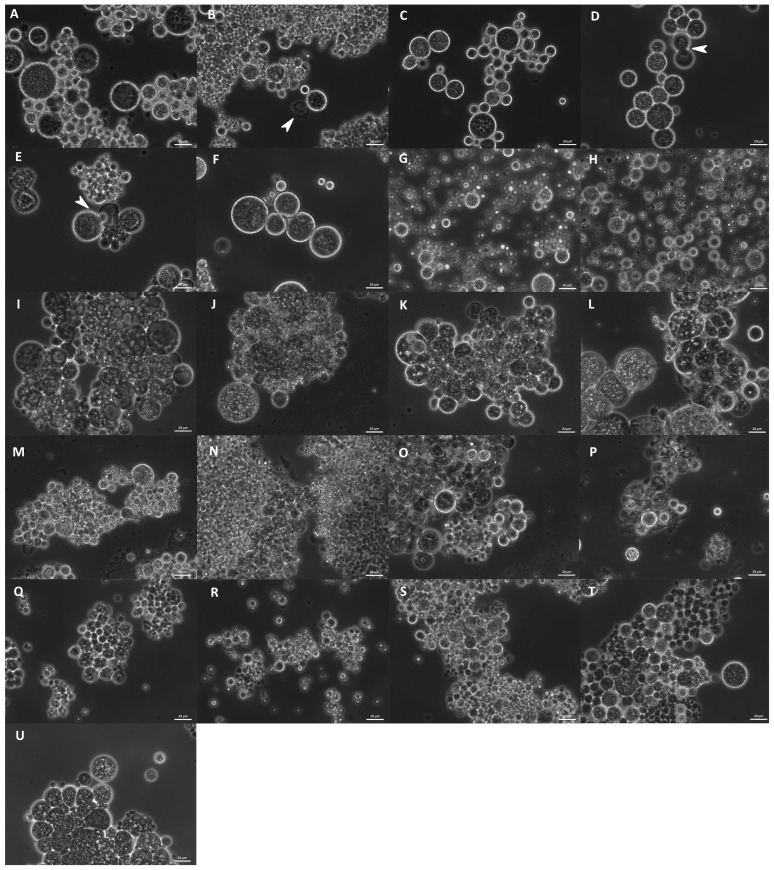
Morphology of various strains observed in B1TMG medium after cultivation in microtiter plates. *U. profunda* N5905 (**A**), N5658a (**B**), *U. visurgensis* 6000b (**C**), Sakar7 (**D**), N5594d (**E**), N5589c (**F**), *T. aggregatum* 4992b (**G**), N4930a (**H**), *S. aggregatum* 561bx (**I**), N2820a (**J**), 5999 (**K**), *T. striatum* 5997 (**L**), *O. minutum* N5995 (**M**), 5996 (**N**), *T. kinnei* N1694d (**O**), 1476c (**P**), *Thraustochytriidae* sp. N5670c (**Q**), N4994d (**R**), *T. aureum* N6006d (**S**), N6007e (**T**), and 5985 (**U**). Arrowheads indicate “hatching” cells and their cell wall remnants. All figures are in identical scale, scale bars show 20 µm.

The studied strains differed in their morphology. Cell size varied from approximately 2 to 80 µm, and different cell shapes were observed. Cell size and form varied not only between the different strains but also changed after the cultivation of the same strain in different media. All strains were compared after growth in B1TMG (2.5 mL), the basal medium and positive control for the subsequent growth and optimization studies. Most strains exhibited healthy cells in this medium (Figure 9). All strains had a wide cell size distribution at all observed growth phases. Strains closely related to *Ulkenia* spp. (Figure 9A–F) showed many globose cells with highly refractive cell walls. In *U. profunda* N5658a and *U. visurgensis* N5594d and Sakar 7, “hatching” cells were observed, i.e., the inner part of a cell slowly left its cell wall through a small spot (Figure 9D,E). The cell wall remained nearly intact. The resulting protoplasts were perfectly globose and did not resemble an amoeba. This behavior was observed in mature and comparably large cells. *T. aureum* N6006d and N6007e (Figure 9S,T) also formed round cells but were more densely packed. Their cell walls were not as refractive as in *Ulkenia* spp. except for some larger cells (>25 µm). *T. aureum* 5985 (Figure 9U) differed from the other two strains with generally larger and more irregular cells. *T. kinnei* N1694d and N1476c (Figure 9O,P) formed more clusters with fewer single cells. The cells of *S. aggregatum* 561bx, N2820a, and 5999 (Figure 9I–K) had a granular sub-structure. *S. aggregatum* 561bx, in particular, showed intracellular bodies. Single cells of *S. aggregatum* were among the largest observed (30–50 µm), and dense clusters of cells with difficult to distinguish and irregularly shaped cells were observed in medium 3. *O. minutum* N5995 and especially 5996 (Figure 9M,N) formed clusters of comparably small cells. In cultures of *T. striatum* N5997 (Figure 9L), the largest cells up to 80 µm were observed. Its cells exhibited finer and coarser substructures and vacuole-like compartments. *Thraustochytriidae* sp. N5670c and N4994d (Figure 9Q,R) were more dispersed and showed smaller agglomerates with smaller cells and cellular structures that might have been small sporangia (10–15 µm). Their substructure was very fine and smooth. All the named strains formed some agglomerates. *T. aggregatum* 4992b and N4930a (Figure 9G,H) differed from them by their very equally distributed small or medium-sized cells compared to the other strains.

When comparing the morphology of strains grown under different nutrient conditions (Figures S3–S6), it was not possible to clearly detect patterns in morphological change between the different media and strains. The formation of agglomerates generally increased in the DoE media. *T. aggregatum* N4930a, 4992b mainly remained dispersed. Strains related to *S. aggregatum* showed clusters of amorphous cells, especially in medium 3. Irregularly shaped cells were more frequently detected in media 3 and 6 compared to the other media for most strains. Some phenomena were observed in the different genera, which might underline their taxonomic peculiarities.

**Figure 10:**
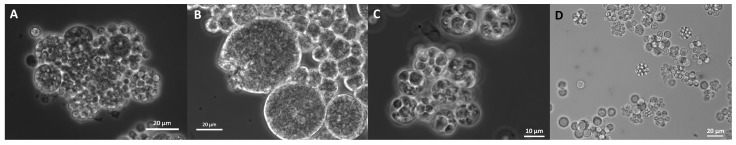
Sporangia in *T. aureum* N6006d (medium 12) (**A**), *T. striatum* N5997 (B1TMG, inoculation culture) (**B**), *Thraustochytriidae* N5670c (B1TMG) (**C**), and N4994d (B1TMG, inoculation culture) (**D**).

In addition to *Ulkenia* spp., “hatching” cells were observed in *T. striatum* N5997 and *T. kinnei* N1694d. These cells were mature and comparably large. Thus, the remaining cell wall remnants were also comparably large (up to 20 µm) and often slightly deformed. Similar empty cell walls were also observed in *T. aureum* N6007e (Figure S5T) but without the phenomenon of “hatching” cells. Microscopic observation suggests the development of sporangia only in a few strains. Few cells containing several spore-like cells were observed (Figure 10), e.g., in *T. aureum* N6006d and N6007e, *S. aggregatum* 561bx, N2820a, and 5999, *T. striatum* N5997, and *Thraustochytriidae* sp. N5670c. However, flagella as proof for zoospores were not observed, possibly due to the resolution. It might be that only aplanospores were built. These sporangia were also observed in the 7-day-old cultures used for inoculation, in *T. striatum* N5997, *T. aureum* N6006d and N6007e, *Thraustochytriidae* sp. N4994d, N4995d, and N5670c, and *U. visurgensis* 5594d.

**Figure 11:**
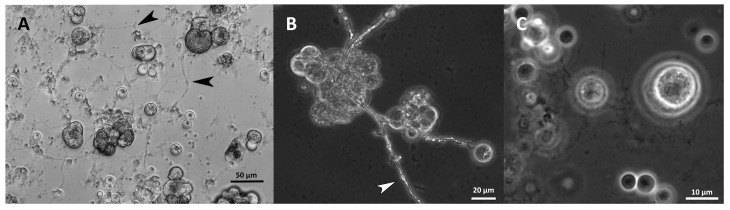
EN with knot-like structures in *S. aggregatum* 561bx (medium 3, analyzed by inverted microscopy) (**A**), and 5999 (medium 12) (**B**), fine EN of *O. minutum* 5996 (medium 12) (**C**). Black and white arrowheads indicate knot-like structures in the net.

Most strains formed an ectoplasmic net (EN) on the bottom of the well. Intact and whole structures of ENs were only visible in the cultures analyzed by inverted microscopy (Figure 11A). It was observed in *S. aggregatum* 561bx, N2820a, 5999, *O. minutum* N5995, 5996, *T. aureum* N6006d, N6007e, *T. kinnei* N1476c, N1694d, and *T. striatum* N5997. Additionally, *S. aggregatum* 561bx, N2820a, 5999, and *O. minutum* N5995 formed a knot-like structure within their EN. Those structures were smaller than any cells in the culture and showed the same light refraction in the microscope as the EN (Figure 11A,B).

**Figure 12:**
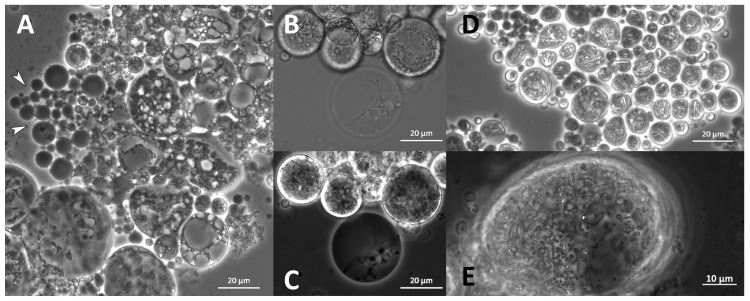
Empty cell structures indicated with white arrowheads observed in B1TMG inoculation cultures of *T. striatum* N5997 (**A**) and *T. kinnei* N1694d in bright-field (**B**) and phase-contrast microscopy (**C**). Oblong cellular substructures in *T. kinnei* 3041c (B1TMG, inoculation culture) (**D**) and together with small granules in N1694d (medium 12) (**E**).

Structures that appeared like “empty cells” or membranes were observed. Their size varied, but they were primarily small (<10 µm) and round without deformations (Figure 12A). They seemed primarily empty but sometimes contained smaller spheres or thread-like structures (Figure 12B,C). They appeared in all analyzed species and most strains with varying abundance. Thin, elongated, and branched cellular substructures were observed exclusively in *O. minutum* N5995 and in the strains that were closely related to *T. kinnei*. They sometimes appeared together with very small, globose granules. The structures were found in medium 12 and, to a lesser extent, in medium 14 (Figure 12E). They also occurred in *T. kinnei* N1709d and 3041c in the cultures used for inoculation of the experiments in B1TMG (Figure 12D).

**Figure 13:**
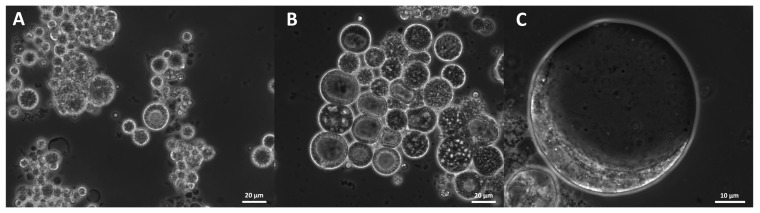
Ring-shaped cell interior of *T. aureum* N6007e (medium 12) (**A**), *U. visurgensis* N5594d (medium 12) (**B**), and crescent-shaped cell interior of *T. striatum* N5997 (medium 14) (**C**).

In some media, the cells exhibited round or oval vacuole-like inclusions, pushing the protoplast to the edge of the cell, resulting in a ring or crescent shape (Figure 13). They appeared in all analyzed species and most strains with varying but primarily low abundance.

**Figure 14:**
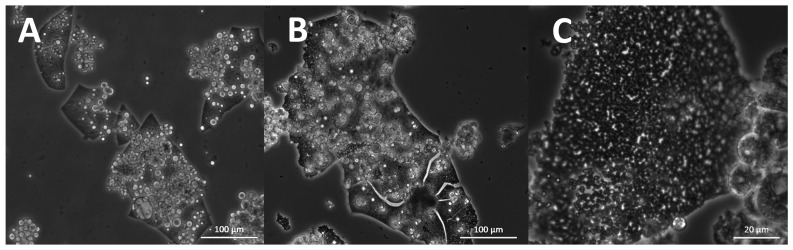
Polymer-like structure in *T. aureum* N6007e (medium 12) (**A**), *T. kinnei* N1476c (medium 3) (**B**), and *T. striatum* N5997 (medium 3) (**C**).

A polymer-like structure was observed (Figure 14). Mostly it floated on the medium surface of some culture wells and was even macroscopically perceptible. This structure consisted of small subglobose grains, which were also visible on the surface of the cells and surrounded them. It was noticed in all analyzed strains related to *S. aggregatum*, *T. aureum*, *T. aggregatum*, *Thraustochytriidae* sp., *T. kinnei*, and *T. striatum*, but only in some related to *O. minutum* (N5995) and *Ulkenia* spp. (*U. profunda* N5658a, *U. visurgensis* N6000b, Sakar7). It occurred predominantly in medium 3 and to a lesser extent in the other DoE media but never in B1TMG.

A further characteristic was observed in all strains related to *T. aggregatum*. They showed a distinct color switch from pale white to brownish-red on some of the solid media of the DoE. The color was media- and time-dependent, as some cultures were initially white and turned red over time, although not in a gradual color change but in a sharp transition.

## 3. Discussion

Uncertain or outdated strain denomination in the literature is a common problem in Thraustochytriaceae research. Many strains are not characterized at species level or are even assigned to the wrong genus. There is high uncertainty, especially regarding the genus *Schizochytrium*. Many of the strains used today still are addressed as members of the genus *Schizochytrium,* although 18S rRNA data reveal a closer relationship to *Aurantiochytrium* sp. than to *Schizochytrium sensu stricto* as proposed by Yokoyama and Honda in 2007 [63]. Accordingly, some strains used for comparison, whose 18S rRNA sequences were obtained from the GenBank database or publications, were reclassified according to their closest relatives, where appropriate. All strains whose 18S rRNA sequences were reviewed by comparison with the dataset are marked with an asterisk in the following text and can be found in Table S12 and the phylogenetic tree (Figure 1), where possible.

### 3.1. Molecular Identification

The strains of the collection were assigned to eight different clades within the Thraustochytriaceae. One group of strains was closer related to the Rhizophydiales. The phylogenetic tree obtained is essentially similar to those in recent publications [63,64,65,78,87,88,89,90,91]. It was divided into two main clades, one containing only Thraustochytriaceae and one comprising Thraustochytriaceae and the Labyrinthulaceae. Strains N557a and N1694d were assigned to *S. aggregatum* and *T. kinnei*, respectively. This corresponds to their previous classification [61,62,92].

### 3.2. Growth Studies

For most strains, glucose and yeast extract concentrations up to the maximum level were considered best for optimal growth. The linear influence of yeast extract was most important, as it was significant for the growth of 93% of the tested strains. This is in agreement with studies for various members of the Thraustochytriaceae. Stefánsson et al. reported in a similar experiment that the influence of the yeast extract was the most positive and only significant factor in their growth model for an isolate (St5) possibly closely related to *T. kinnei* [87]. *T. aureum* ATCC 34304 was reported to show highest growth at maximum yeast extract (2.5 g/L), peptone (2.5 g/L), and glucose (30 g/L) concentration [93]. Previous studies from Bahnweg, who worked in part with strains of the former KMPB collection, indicated higher nitrogen levels in the form of L-glutamate to generally increase the maximum yield of various strains and genera [92]. Optimal growth for most strains studied here was predicted at the highest yeast extract concentration of 15 g/L used in the experiments, indicating that their optimum might be even higher. In a model developed for *Aurantiochytrium* sp. UMACC-T023*, the sum of maximum yeast extract (20 g/L) and peptone (20 g/L) concentrations was considered optimal for high growth. However, here the glucose concentration was considered best for growth at its minimum of 10 g/L [94]. In this study, a significant influence of the glucose concentration on growth was observed for nearly 75% of the strains. Maximum glucose concentrations of up to 60 g/L were predicted to be optimal for most of the strains. Again, the fact that this was the highest concentration used and the insignificance of the quadratic term in the model for glucose concentration indicated that the optimum growth of the strains might be achieved well above 60 g/L glucose. The aforementioned isolate St5 was shown to reach optimal biomass at a glucose concentration of 77.6 g/L [87] and *Aurantiochytrium* sp. ONC-T18* at 60 g/L [95]. *Schizochytrium* sp. G13/2S still grew well between 100 and 200 g/L [96]. Gupta et al. reported optimum growth of *T. aureum* AMCQS5-5* when cultivated in media containing 40 g/L glucose, though no clear change between 5 and 100 g/L [74].

The C/N ratio was shown to be important, and the highest growth was achieved at C/N between 2 and 4 [74]. Similarly, a low C/N ratio of 5 was advantageous over higher ratios for the growth of *A. limacinum* ICTSG-17* [19] and *Aurantiochytrium* sp. ATCC 26185* [97]. Comparably high C/N ratios of 27.2 and 54.4 increased the growth of *Aurantiochytrium* sp. ATCC PRA-276 as opposed to a ratio of 4 [97]. These differences show that an optimal C/N ratio is possibly very strain-specific and the optimal value also seems to depend on the nitrogen source [74]. In these experiments, the C/N ratio was not regressed, but most of the strains evaluated by regression model two favored high glucose and yeast extract concentrations, indicating a positive influence of a comparably lower C/N ratio.

Thraustochytrids appear to be euryhaline. Optimal salt concentration for growth varied between 2.0% and 2.5% NaCl in various Thraustochytriaceae [92,98] or 25 and 30 practical salinity units (PSU) in several strains of *Aurantiochytrium* sp. [99]. *T. aureum* grew best between 15 and 20 g/L salt concentration [93,100,101]. *Aurantiochytrium* sp. ONC-T18* grew best on a lower salt concentration of 2 g/L [95]. The highest cell dry weight in *A. limacinum* ICTSG-17* was reached in a medium prepared with seawater, but the growth did not decrease much with lower salinity. It was even able to grow at 0% [19]. Other authors showed that growth was inhibited by very low salt concentrations [102]. Few strains, which belonged to *T. aureum* and *S. aggregatum,* were able to grow at 0.1% NaCl and not very well [92]. In contrast, several Thraustochytriaceae grew optimally at salinity levels between 3.5 and 4.2% and survived even higher concentrations [102,103]. Our results revealed no significant difference in growth of most strains depending on a salinity between 1.5% and 3.0%. Only some species belonging to the genus *Ulkenia* showed a significant need for a higher salt concentration. Bahnweg did not show this for an *Ulkenia* strain [92], but this might be species and strain dependent. Adaption to the condition might also influence growth (see below).

The influence of the initial pH was not considered significant for most strains in the tested range between pH 6.5 and 7.6. This is similar to results of Stefánsson et al., who found a positive but insignificant effect of the pH on the growth of their isolate St5 between pH 6 and 8 [87]. The highest biomass of *A. limacinum* ICTSG-17* was measured in a pH range of 6–7 [19]. In several other strains of *Aurantiochytrium,* the optimum was between 6.5 and 7.5 [99]. Bahnweg observed optima between 6.0 and 8.0 for various Thraustochytriaceae strains [92]. Some authors mentioned an increase in pH in a pH-uncontrolled environment [19,104], which could be confirmed here.

The addition of phosphate (KH_2_PO_4_) did not influence the growth of the strains significantly. Its effect was not uniform either. Stefánsson et al. reported a negative but insignificant influence of the KH_2_PO_4_ concentration between 0 and 0.1 g/L on the growth of their isolate [87].

A replacement of the media components might further enhance growth. For example, glycerol improved the growth in various Thraustochytriaceae [74,105]. Optimal nitrogen sources were discussed. Those of marine origin were reported to increase growth compared to yeast extract and tryptone [87]. Temperature and light influenced growth as well [92,99,103]. Various Thraustochytriaceae were shown to have a quite wide temperature range (9–30 °C), except for some Antarctic isolates [92].

Regression quality of the growth data of some strains and genera was limited. Insufficient adaptation of the strains to the new media might have biased the data. All DoE trials were inoculated from the same liquid medium, so the strains had to adapt to some more than others, changing their ability to grow and produce metabolites. The change of medium solidity also influenced growth at otherwise constant parameters. For example, *U. profunda* N5905 did not grow on the solid media with a low (15 g/L) salt concentration but in the same media provided in the liquid form prepared for the morphological observations. It was inoculated from a stock medium with a salt concentration of 30 g/L, and the shock of low salt concentration and solid medium was possibly too high. In contrast, it was able to adapt to the liquid environment. It was observed (data not published) that a rapid change in medium composition and solidity inhibited the growth of some strains, whereas they were able to adapt to more gradual changes, especially if grown in liquid media. We assume that the thraustochytrids can adapt to various environmental conditions if given the chance.

Screening many novel isolates and strains with varying characteristics and needs is challenging. Reduced factorial design of experiments combined with regression analysis was a valuable tool for approaching the strain collection. An impression of growth-promoting parameters was obtained. For a robust regression and to increase accuracy, a sufficient number of experiments must be performed to also evaluate the influence of interaction terms.

### 3.3. Comparison of Growth Analysis and Taxonomy

The screening was designed to determine and compare the growth behavior of possibly closely related strains. As nearly all strains preferred the media with high glucose and yeast extract concentration, a distinction was difficult. Coherent deviating behavior was found in both analyzed strains of *U. visurgensis:* Sakar 7 and N6000b, and of *T. aggregatum:* 4992b and 154f. They were closely related, showed intermediate glucose optima and, with the exception of Sakar7, intermediate yeast extract optima. Strains N5905, N5976, N5658a, and N5629e were closely related to *U. profunda* based on the molecular taxonomy. N5905, N5976, and N5629e did nearly not grow on solid media with a salt concentration of 15 g/L. When the medium was supplied with 30 g/L salt, N5905 and N5629e yielded the highest biomass on media with low or very low glucose and yeast extract concentrations. Similarly, *U. profunda* N5658a had comparably low glucose and yeast extract maxima. All these strains were closely related, indicating unique characteristics of *U. profunda*.

Most strains yielded mean biomass yields of below 2 g/L. *T. aureum* N6006d, N6007e, and 5985, as well as *T. aggregatum* 4992b, 154f, and *T. striatum* N5997 yielded higher mean biomasses of above 4 g/L. The strains belonging to *T. aureum* and *T. striatum* were more closely related to each other than to *T. aggregatum* based on the 18S rRNA phylogeny. *T. aureum* N5998, which was identical to N6006d and N6007e based on its 18S rRNA genes, yielded a much lower medium biomass of below 2 g/L. Thus, characteristics may be very strain specific. Based on the obtained growth results, a precise and well-distinguished differentiation of all the tested strains is difficult. The growth parameters are not suitable to support the taxonomic classification.

### 3.4. Carotenoids

The production chain of carotenoids in thraustochytrids was proposed to include *β*-carotene, *β*-cryptoxanthin, echinenone, hydroxyechinenone, canthaxanthin, zeaxanthin, phoenicoxanthin, adonixanthin and astaxanthin [21,80,83]. The following carotenoids were found in species of the Thraustochytriaceae: astaxanthin, canthaxanthin, echinenone, *β*-carotene, and rarely also lutein, zeaxanthin, lycopene, and astaxanthin esters and isomers [21,74,81,82,97,106,107,108]. Although there are few comparative datasets for the carotenoid patterns of thraustochytrids [63,64], individual strains have been described.

Astaxanthin, phoenicoxanthin, echinenone, and *β*-carotene have been reported in *Ulkenia* sp. SEK 214* and ATCC 28207* [64]. This is similar to the carotenoids observed in these experiments in all strains closely related to *Ulkenia* species. Additionally, small proportions of lycopene and an unknown carotenoid were observed in *U. profunda* N5658a.

*Schizochytrium sensu stricto* (e.g., *S. aggregatum* ATCC 28209*, *Schizochytrium* sp. SEK210*, *Schizochytrium* sp. SEK 345*) was reported to form light yellow colonies with *β*-carotene only [63]. This is in general agreement with the results obtained for the strains closely related to the named species. However, besides *β*-carotene, small proportions of lycopene and an unknown carotenoid were observed. The strains produced a total carotenoid content of up to 49 µg/g.

*T. aureum* was reported to contain up to 44 µg/g of carotenoids but without further differentiation [109]. *T. aureum* AMCQS5-5* and AMCQS5-3* produced primarily canthaxanthin, followed by echinenone and *β*-carotene with a total of up to 68.5 µg/g and no astaxanthin [74]. This is largely consistent with the results obtained in this study. Here, mainly canthaxanthin and *β*-carotene but also echinenone were observed. A total carotenoid content of up to 346 µg/mg was measured depending on the medium.

There was also little information on carotenoids in strains closely related to *Thraustochytriidae* sp. N4994d, N4995d, N5670c, such as *Labyrinthulochytrium* spp., whose colonies were reported to be grayish white [110,111], possibly depending on the medium. Strains N4994d, N4995d, and N5670c were also colorless on some DoE media. Another related strain found was *Phycophthorum* sp. RT2316-16* [112]. It contained mainly canthaxanthin (86.5–87.4%), followed by astaxanthin and *β*-carotene in similar proportions, depending on the cultivation conditions [106]. It reached a total carotenoid content from 64 to above 200 µg/g [106,112,113]. Canthaxanthin also had the highest proportion of carotenoids in *Thraustochytriidae* sp. N4994d, N4995d, and N5670c in the form of all-*E*-canthaxanthin and 9*Z*-canthaxanthin, followed by astaxanthin and phoenicoxanthin, indicating a possible relationship to *Phycophthorum* sp. RT2316-16*.

The analyzed carotenoids in *T. striatum* N5997 were mainly astaxanthin (mean of 85% in total), followed by significantly lower proportions of phoenicoxanthin, canthaxanthin, lycopene, *β*-carotene, and two unknown components. *T. striatum* N5997 had a mean carotenoid content of 42 µg/g in the DoE and a maximum content of 69 µg/g (medium 15). Yokoyama et al. [63] analyzed *T. striatum* ATCC 24473* and found the same carotenoids except for lycopene. In the same strain, up to 600 µg/g astaxanthin under pH stress conditions was reported. Additionally, zeaxanthin, canthaxanthin, echinenone, *β*-cryptoxanthin, and *β*-carotene occurred [114]. In *T. striatum* AL16*, mainly astaxanthin and zeaxanthin, and up to 12 µg/g of total carotenoids were detected [115]. Singh et al. analyzed *T. striatum* S7* and reported astaxanthin (67 µg/g) as main carotenoid, followed by canthaxanthin (20µg/g), echinenone (17 µg/g), and *β*-carotene (11 µg/g) [81].

Yokoyama et al. observed canthaxanthin, echinenone, and *β*-carotene in *Oblongichytrium* sp. SEK347* [63], but no carotenoids were measured in *O. minutum* N5995 and 5996.

Astaxanthin, phoenicoxanthin, canthaxanthin, echinenone, and *β*-carotene were identified in *Aurantiochytrium* sp. ATCC 26185* and *Aurantiochytrium* sp. ATCC PRA-276 with maximum values of 77 and 180 µg/g total carotenoids, respectively. Their carotenoid profiles varied with the culture conditions [97]. Burja et al. measured *β*-carotene, echinenone, canthaxanthin, zeaxanthin, and astaxanthin in their strain *Aurantiochytrium* sp. ONC-T18* [95]. Major carotenoids in *Aurantiochytrium* sp. S31* (ATCC 20888) were either *β*-carotene and astaxanthin [104] or astaxanthin, canthaxanthin, and echinenone [74]. In *Aurantiochytrium* sp. AMCQS1-9*, only *β*-carotene was reported [74], although phylogeny implied a close relationship to Aurantiochytrium* sp. S31. Astaxanthin, phoenicoxanthin, canthaxanthin, echinenone, and *β*-carotene were observed in *Aurantiochytrium* CHN-1* with a total of almost 450 µg/g [82].

The genus *Aurantiochytrium* seems very potent concerning carotenoid production, although the reported results vary. This clade might be much larger than previously assumed and is currently not very well structured. A clearer organization of the contained species and strains and carotenoid production under coherent conditions need to be achieved for further insight into the applicability of chemotaxonomy in this clade. Meanwhile, other thraustochytrids may be underexplored concerning their carotenoid synthesis potential. *T. aureum* und *Thraustochytriidae* sp. had the highest mean measured carotenoid content and comprised the strains with the highest carotenoid content of all on individual media: *T. aureum* 5985 produced 346 µg/g of carotenoids (medium 15) and *Thraustochytriidae* sp. N4994d 300 µg/g (medium 6). Most strains belonging to *T. aureum* produced comparably high biomasses, and thus the total carotenoid production was high. The clade of *T. striatum* might also contain good carotenoid production strains. As indicated by comparison with the literature, exact yields might be strain specific but also dependent on the culture conditions and may be improved.

#### 3.4.1. Cluster Analysis of Carotenoid Composition

Cluster analysis of carotenoid composition detected nine main carotenoid clusters. *U. profunda* N5905 and N5629e carotenoid data were limited to salt condition media, as these strains grew mainly on media with a high salt concentration. The dendrogram obtained by hierarchical clustering mirrored k-means clusters and added a level of detail. The k-means clusters were comparable to the clades created by the phylogenetic tree based on 18S rRNA gene sequences.

Direct comparison of the clusters based on the carotenoids with the phylogenetic tree revealed high similarities. All strains closely related to *Thraustochytriidae* sp. were clustered, as well as those belonging to *Ulkenia* spp., *S. aggregatum*, and *T. aureum*. *T. striatum* N5997 was placed close to the *Ulkenia* species, which is also evident in the phylogenetic tree. Its phylogenetic relationship to *Thraustochytriidae* sp. was not displayed by the dendrogram based on carotenoid patterns. The division of the *Ulkenia* species into different subgroups based on their carotenoid profiles is generally similar to the 18S rRNA data. Adding the strains that only grew on a higher salt concentration increased the robustness of the clustering. *U. profunda* N5658a, N5905, and N5629e formed one clade in both dendrograms. In *U. visurgensis,* two slightly separated clades (Sakar 7 and N6000b/N5594d and N5589c) were identified by both methods. A difference was the separation of *T. aureum* N5998 from the other members of this group, especially from N6006d and N6007e. These three strains were taxonomically identical on an 18S rRNA basis.

On a greater scale, strains belonging to *Thraustochytriidae* sp. and *T. aureum* built one larger clade based on carotenoid profiles, and *T. striatum* N5997 clustered close to *Ulkenia* spp. However, in the molecular phylogeny, *Thraustochytriidae* sp. was most related to *Ulkenia* spp., which together shared a common ancestor with *T. striatum*. *T. aureum* was least related to all of them. *S. aggregatum* species were clearly distinct from all other clades based on 18S rRNA and carotenoid-based taxonomy.

Carotenoid patterns might support phylogenetic studies and help assign strains to a particular genus or even species. However, chemotaxonomic markers alone cannot explain the relationship between the different clades. Fossier Marchan et al. [116] stated in their review that the genus *Thraustochytrium* does not form a monophyletic group and that no common carotenoid profiles were observed. We agree with the first part and argue that the phylogenetic classification of not only the genus *Thraustochytrium,* but also the entire family of Thraustochytriaceae needs a comprehensive and coherent revision. However, we also conclude that the carotenoid profiles are coherent enough to provide chemotaxonomic support to an 18S rRNA gene and morphology-based approach. Carotenoid compositions of individual strains of a species were similar, but the detailed composition changed depending on the medium. Thus, an agreement on how to collect such data must be reached. The detailed individual carotenoid composition and yield of a strain, and to some extent its growth behavior, might help to further distinguish strains with highly similar 18S rRNA sequences, providing an approach for a polyphasic taxonomy.

#### 3.4.2. Carotenoid Regression

Strain *T. striatum* N5997 was characterized by a relatively constant carotenoid production on most DoE media. Therefore, it was chosen for closer identification of carotenoid production parameters. The optimized regression for growth showed a high affinity for glucose but low yeast extract concentration. Shene et al. showed the highest growth in *T. striatum* AL16* at a maltose concentration of 60 g/L compared to lower concentrations [115]. In *T. striatum* ATCC 24473*, the highest biomass was yielded between glucose concentrations of 30 and 40 g/L and 4 and 20 g/L yeast extract and peptone [114,117]. The growth of *T. striatum* N5997 was even inhibited by high yeast extract concentrations.

Maximum content of most carotenoids was predicted at high glucose (48.4–60 g/L) and minimum yeast extract concentration (0.5 g/L), at maximum pH (6.7), and maximum phosphate addition (0.5 g/L). Similarly, higher carotenoid yields in *T. striatum* AL16* at increasing maltose levels were demonstrated [115]. Xiao et al. demonstrated increasing astaxanthin content in the cells using either maximum glucose concentration (100 g/L) or low yeast extract and peptone concentration (2–4 g/L) in *T. striatum* ATCC 24473*. They also showed that astaxanthin yield decreased at glucose concentrations higher than 50 g/L because of decreasing cell mass [114]. This was not shown here, possibly because the experiments were limited to glucose concentrations of 60 g/L. A high carbon-to-nitrogen (C/N) ratio seemed favorable for a high carotenoid content.

The carotenoids changed depending on the C/N ratio in *Aurantiochytrium* sp. S31* (ATCC 20888). Particularly high (75:5) and low (75:30) C/N ratios resulted in high carotenoid content (85–89 µg/g). The high ratio also caused a shift in the carotenoid pattern in favor of astaxanthin, the proportion of which exceeded that of *β*-carotene [104]. Such a drastic change in carotenoid composition was not observed in our experiments. Furlan et al. [97] reported that generally lower C/N rations favored a high carotenoid concentration in batch cultures and that changes in cultivation conditions impacted the carotenoid profiles of *Aurantiochytrium* sp. ATCC 26185* and ATCC PRA-276. Increased production of carotenoids and fatty acids under stress conditions like nitrogen starvation is also known in various microalgae [118]. Like in microalgae, carotenoid production in thraustochytrids seems to be correlated with the production of fatty acids [113], and factors influencing fatty acid production are likely to influence carotenoid production as well.

Increasing KH_2_PO_4_ concentration (up to 0.5 g/L) was reported to have a beneficial influence on the carotenoid concentration of *Aurantiochytrium* sp. S31* and also to change the proportion of individual carotenoids [104]. The prediction for optimal carotenoid content and yield in *T. striatum* N5997 indicated the maximum phosphate level as favorable.

Carotenoid production might be further improved, and carotenoid profiles might be manipulated by the replacement of, e.g., the carbon source [74,104,106,115]. It also depends on environmental factors such as temperature and aeration and is likely to be influenced by light [19,21,114]. Overall, the carotenoid pattern varied with changing medium composition. Such a varying composition was also reported in *T. striatum* S7* and other Thraustochytriaceae during cultivation [81,82,83,112]. Finding the optimal time for harvesting is another approach to maximizing carotenoid yield.

### 3.5. Squalene

Squalene has been observed mainly in *Aurantiochytrium* sp. [69,70,71,119,120,121,122,123], and to minor extents in *Schizochytrium* sp. ACEM 6063 [124], strains closely related to *T. aureum*, *T. striatum*, *Oblongichytrium* sp., *Parietichytrium* sp., *Botryochytrium* sp., and *Ulkenia* sp. [120]. Squalene was found in all strains analyzed in this study. Thus, the majority of the family of Thraustochytriaceae might be able to produce squalene under certain circumstances. The highest squalene contents observed were 13 mg/g and 12 mg/g in *T. striatum* N5997 on medium 11 and *O. minutum* N5995 on medium 16 with a high salt concentration, respectively, and 10.4 mg/g in *S. aggregatum* N2820a on medium 6. This is less than the 317 mg/g found in *Aurantiochytrium* sp. [120]. Still, it might be worth examining other members of the Thraustochytriaceae for squalene production and optimizing cultivation parameters further, especially as growth and biomass density are equally important for high total yields, as also Aasen et al. [80] stated. To our knowledge there has been no description of squalene in *T. kinnei*, and *T. aggregatum*. The results show that even within clades of closely related species, the variance in productivity may be high. High differences in squalene production by closely related strains belonging to *Aurantiochytrium* and *Hondea* were described [125].

Studies showed that squalene synthesis depends on the nitrogen source and that yeast extract concentrations of 2.5 or 6 g/L were optimal concerning squalene content and yield in *Aurantiochytrium* sp*. [121,126]. However, evaluating such values in isolation from other parameters is difficult. A mixture of different nitrogen sources positively influenced squalene content and yield in Aurantiochytrium* sp. BR-MP4-A1 [121]. The model developed for *T. striatum* N5997 generally implied a higher yeast extract concentration of 15 g/L as beneficial for high squalene content. However, such a high yeast extract concentration did not increase the biomass yield, and thus optimal squalene yield was estimated at 8.2 g/L yeast extract. The opposite correlation was shown for the glucose concentration. Here, the squalene content was predicted to be high when no glucose was provided in the medium, but growth was low. This contrasts studies that found glucose concentrations of 20–30 g/L to be optimal for squalene yield and content in *A. mangrovei* and *Aurantiochytrium* sp.* [79,126]. Again, a cross-genus comparison must be considered with caution.

There are also contrasting studies about the temporary course of squalene formation. Squalene content was reported to increase over 1 to 8 days of the experiment [122] or to decrease slightly between day 4 and 12 [119]. Other studies showed a rapidly decreasing squalene content after the initial stage of cultivation [79,127], which was correlated to lipid accumulation [127]. Increased squalene and carotenoid content were reported in *A. limacinum* B4D1* by adding methanol or butanol, which was correlated to a change in lipid composition, especially a decrease in docosahexaenoic acid (DHA) [108,128]. Squalene is the first step in sterol synthesis [124] and necessary for membrane building. Squalene synthesis was correlated to other factors such as temperature, carbon and nitrogen source, dissolved oxygen, and NaCl concentration [124,126], some of which also impacted the lipid profiles [129]. Squalene synthesis is very likely connected to fatty acid and carotenoid synthesis. A deeper understanding of the interrelations and corresponding optimization could improve productivity even further. The activation and inhibition of certain enzymes in the metabolic pathways of squalene is another approach to improving its production [79,130]. It is likely that optimal conditions regarding relative and total squalene productivity also vary between the different species of the Thraustochytriaceae, as seen in the different literature results.

### 3.6. Comparison of Growth and Target Molecule Synthesis on Solid and in Liquid Medium

Several strains were tested on their biomass, squalene, and carotenoid productivity in liquid and solid cultures. In liquid culture, the maximum cell density achieved was 1.1 g/L in *Thraustochytriidae* sp. N4994d. Between 4.0 and 7.9 g/L was reported for *T. aureum* ATCC 34304 [93,100,131,132,133]. Cultivation of *Aurantiochytrium* sp.* yielded biomass densities between 6.25 and 27 g/L [94,134,135,136,137] but also up to 154 g/L [72]. Generally higher biomass densities of up to 65–200 g/L and productivities of around 5–8 g/L*h were reported for *A. limacinum* and *Schizochytrium* sp. [84,85,96]. Other genera of the Thraustochytriaceae, e.g., *Ulkenia*, *Oblongichytrium*, *Botryochytrium*, and species such as *T. kinnei* and *T. aggregatum* have been scarcely studied for their productivity. For the strains studied here (N4994d, N6006d, N5658a, and N1001), the biomass density on solid medium was higher than in liquid culture. On solid medium, a maximum yield of 1.8 g/L was reached (*T. aureum* N6006d on medium 7). The best-growing strain in the DoE yielded 27 g/L (*T. aureum* N6007e on medium 8). The higher productivity and density of thraustochytrids on solid media might be correlated to their natural behavior in the marine environment. They build biofilms and decompose marine detritus, and increasing cell densities were measured on decaying matter [138,139,140]. A biofilm-based cultivation approach might be useful to enhance productivity, reduce media volumes, space for cultivation, and thus increase production efficacy. Such cultivation designs have already been proposed for microalgae cultivation [141,142,143,144].

The production of the target molecules depended on the state of the medium. Generally, more carotenoids were produced on solid medium but less squalene and vice versa. Moreover, the carotenoid profile of the strains changed. A solid medium might also be a stress factor because the availability of nutrients and oxygen availability is limited to diffusion. The close proximity of the cells might induce competition for resources and space. Lower water availability and air exposure might lead to dry stress. An increased production of carotenoids might be a countermeasure.

### 3.7. Effect of the Medium Composition on the Metabolites

Comparison of the target molecule content of selected strains on three different media (DoE 6, 9, and 15) showed that for most strains, the highest carotenoid contents were yielded in the medium with high glucose and low yeast extract concentration (medium 15). The squalene content was generally lower under these conditions, as predicted by the carotenoid regression and optimization of *T. striatum* N5997. In contrast, squalene and carotenoid content in the medium with low glucose and yeast extract concentration (medium 6) were higher and lower, respectively. Thus, conditions that support squalene synthesis obstructed carotenoid synthesis and vice versa. *Thraustochytriidae* sp. N5670c and N4994d were an exception to this pattern as the highest measured carotenoid levels were observed in medium 6, indicating that exceptions to that rule are possible.

Medium 15, which favored the production of carotenoids, generally also influenced the extract yield positively, indicating a positive correlation between fatty acids and carotenoid synthesis. The highest extract yields were obtained from *T. aureum* N6006d and 5985. The fatty extracts show that these strains from this clade might be used as a co-producer of fatty acids and carotenoids. Various authors already revealed a high fatty acid and docosahexaenoic acid content in *T. aureum* [93,100,131,132,133,145].

Farnesyl pyrophosphate is a starting point for the synthesis of carotenoids, and squalene and sterols. Likewise, the common precursor acetyl-CoA is also the starting point for fatty acid synthesis [80,108]. Environmental and nutritional conditions might induce a switching between all those pathways. In addition, carotenoid patterns changed depending on the medium composition, showing that understanding the underlying mechanisms is crucial for exact predictions.

Evaluation has shown that carotenoid synthesis in thraustochytrids was dependent on the strain and the medium composition. Strains belonging to *T. aureum* and *Thraustochytriidae* sp. synthesized a high carotenoid content and might be promising genera for biotechnological carotenoid production. However, in *T. aureum,* large differences between some of the strains were observed. Strains belonging to *S. aggregatum* produced relatively more squalene than the other strains.

It seemed that carotenoid and squalene production somehow canceled each other, both in terms of their dependency on opposing medium composition and condition and depending on the production capability of the strain. Most strains that produced higher amounts of squalene did not synthesize high amounts of carotenoids and vice versa. *Ulkenia* spp. and *T. striatum* represented a compromise between squalene and carotenoid synthesis, but *T. striatum* yielded higher biomass.

### 3.8. Unknown Metabolites

Unknown substances with absorption properties in the visible spectrum were detected, and some of them were assigned to further carotenoids: Unknown 1 had a similar absorption spectrum and mass to micromonal [1,146], but this carotenoid was described in the green algal order Mamiellales [147] but not in thraustochytrids. Unknown 2 and 3 had a UV/Vis absorption spectrum similar to echinenone and adonixanthin [1,148]. In unknown 2, the mass peak at *m*/*z* 566.4 resembled the exact mass of 2- or 3-hydroxyechinenone of *m*/*z* 567.42 [M+H]^+^. The second mass peaks at *m*/*z* 549.8 might have resulted from cleavage of the hydroxyl group and is similar to the exact mass of echinenone of *m*/*z* 551.43 [M+H]^+^. 2 and 3-hydroxyechinenone have a similar λ_max_ to echinenone [1,148]. 3-hydroxyechinenone and 3′-hydroxyechinenone were described as intermediates in the astaxanthin synthesis pathway in vitro [149], which was accepted and adapted for cyanobacteria and thraustochytrids [83,148]. Because of similarities of the masses, unknown 2 might be 3-hydroxyechinenone or 3′-hydroxyechinenone but cannot be further differentiated. Unknown 3 might be either adonixanthin or an enantiomer or constitutional isomer of hydroxyechinenone, but it is questionable because of missing reliable mass data. It was excluded that these substances were diastereomers of echinenone, since the retention times of the *Z*-forms are usually longer than that of the all-*E*-form. The occurrence of these substances is plausible because they are part of the carotenoid metabolism. Unknown 4 had a characteristic double peak with a shoulder. It was similar to those of γ-carotene and rubixanthin [1,148,150]. Due to its late retention time and the fact that γ-carotene might be part of the carotenoid synthesis chain as in *Xanthophyllomyces dendrorhous* [151,152], it is more likely to be γ-carotene.

One group of substances with similar and very characteristic UV/Vis absorption spectra was only present in *O. minutum* N5995. Unknown C is possibly protoporphyrin IX. Its main observed mass was *m*/*z* 563.33, which is similar to the mass of protoporphyrin IX with *m*/*z* 563.27 [M+H]^+^. Their absorption spectra were also similar [153,154,155]. It was accompanied by smaller peaks with similar absorption spectra, which were possibly also porphyrins [156,157,158].

### 3.9. Morphology

The different Thraustochytriaceae were heteromorphic. One major characteristic of the genus *Ulkenia* is its amoeboid cell stage. Its cell wall either disappears or the protoplast leaves the cell wall through a small opening [64]. This was observed in strains closely related to *U. visurgensis* and *U. profunda*. It was described that species belonging to the genus of *Ulkenia* only formed small colonies [64], which was basically confirmed here. *Ulkenia* was also characterized by a highly refractive cell boundary in these experiments. It has been described in the literature as having a discrete cell wall, which was thin during growth, but also thick membranes were observed [64,159,160]. *T. aureum* was described to form large conglomerates [161] with globose or subglobose cells [101] and diameters up to 17 [101] or 65 µm [161]. Except for the larger diameters, these observations were confirmed here. *Schizochytrium aggregatum* forms cell clusters and globose cells with diameters between 6 and 12 µm [162]. These may grow by its special ability to perform successive binary division [63] and were described to end in an amorphous mass by Goldstein and Belsky [162]. In this experiment, *S. aggregatum* 561bx, N2820a, and 5999 formed large cell clusters, sometimes with larger individual cells than reported in literature. In some media, mainly number 3, bipartition was observed by the appearance of such amorphous cell clusters. In B1TMG, intracellular bodies were observed in all three strains. These might have been the described vacuole-like structures or lipid bodies. *Schizochytrium sensu lato*, especially *Aurantiochytrium*, is known for its high fatty acid content [163]. Reports about fatty acids in *S. aggregatum* are scarce, nevertheless they have been described, although not in high amounts [132,164]. Yokoyama and Honda described that *Oblongichytrium* sp. formed large cell clusters and was characterized mainly by the ellipsoidal form of its zoospores [63]. In these experiments, *O. minutum* N5995 and 5996 also formed large cell clusters, but no zoospores were observed. Both strains, especially 5996, were characterized by their generally small cells. *T. kinnei* (N1476c, N1694d) formed large cell agglomerates, whereas almost no cell clusters were observed in *T. aggregatum* (N4930a, 4992b) and only very few in *Thraustochytriidae* sp. (N5670c and N4994d). The older studies on the Thraustochytriaceae, which investigated *T aggregatum* [160,165] among others, were mostly performed with pollen cultures, which are not comparable to the experimental design here. Goldstein already summarized the problem of varying morphology under different culture conditions in 1973 [166].

Residual cell walls were found in several strains and genera. The literature distinguishes between two types of persistent cell walls: the cell wall that remains after the release of a protoplast and the persistent cell wall after the release of zoospores. The remaining cell wall after protoplast release was reported in *Ulkenia.* sp. [64], which was also observed in this study. Likewise, at least some of the cell walls observed in *T. striatum* N5997 are likely due to this phenomenon. All described protoplasts were perfectly round and indistinguishable from other cells once they completely had left the cell wall. The amoeboid form described for *Ulkenia* spp. and *T. striatum* [64,167,168] was not observed. In *T. striatum*, disappearing and persistent cell walls after the release of zoospores were reported [169,170,171]. Therefore, the observed walls might be attributed to the release of protoplasts and spores. Empty cell walls in the cultures of *T. aureum* N6007e and *T. kinnei* N1476c might also be persistent sporangial walls like described for *T. aureum* and *T. kinnei* [101,172,173]. In *T. kinnei*, hatching cells were observed, which has not yet been described in the literature.

Sporangia were observed only rarely in most strains. Exceptions were *Thraustochytriidae* sp. N5670c and N4994d but with very small sporangia. Their closest relative in the phylogenetic analysis was *Labyrinthulochytrium* spp. To date, only two strains, *Labyrinthulochytrium arktikum* and *haliotidis*, have been described. They reproduced by binary division and formed even smaller sporangia (7.8–8.9 µm) than observed here, containing three to eight zoospores [110,111].

Zoospores were not observed at all. According to Goldstein and Belsky, zoospores keep their swimming motility for 15 min to 3 h after their release from the zoosporangium [162]. Iida et al. reported that they saw zoospores only in the early growth phase of *T. aureum* but not anytime later [100]. It is therefore likely that the timing of microscopy (13 days after inoculation) was too late. Thraustochytriaceae can also produce aplanospores, just like *Aplanochytrium* [67,174]. So, it is possible that under the given conditions, only aplanospores were released, which would explain the very small cells in some of the cultures.

Structures that resembled smaller “empty cells” floated freely in the medium of most strains. However, their refraction was slightly different, they were perfectly round and closed, whereas the previously described cell walls often were irregularly shaped and damaged.

Generally, Thraustochytriaceae are known to form an ectoplasmic net (EN) [63,64,67,161,174,175,176], which could be confirmed. Compared to ENs in the literature, the nets obtained in these experiments seemed underdeveloped, which might be attributable to the nutrient-rich media. ENs play a part in digestion and nutrient intake [176,177,178]. They were finer when no food source was present, and nutrient concentration in the medium was high [175]. Knot-like features in the ENs of *Schizochytrium* and *Oblongichytrium* were observed. Iwata and Honda described that the EN of *Schizochytrium* thickens once it attaches to a food source [175]. Similar knot-like features have been recorded in *T. striatum*, *T. kinnei,* and *Aplanochytrium* sp., but the authors did not describe the structure [67,176]. In strains related to *T. kinnei* and *O. minutum*, oblong, branched cellular substructures were observed. Weete et al. documented similar structures in *Aurantiochytrium* sp. ATCC 26185*. They discussed that these structures were associated with lipid bodies and lipid synthesis and showed that they became internalized in the growing lipid bodies [179].

Vacuole-like substructures were observed in different strains. Similar compartments were described in *Schizochytrium sensu stricto*, *Oblongichytrium*, *Aurantiochytrium*, and *Mucochytrium quahogii*. However, the authors could not define their function [63,180,181].

Taxonomy based on morphology only was not possible in this group. Although strains from the same clade had a similar appearance even under different medium conditions and sometimes showed very specific characteristics, cross-clade assessment was difficult. Strains that were only distantly related shared common traits, which were not present in closer relatives. Moreover, the expression of many features depended on the medium, but the variability of different strains under different conditions also aggravated classification.

### 3.10. Synopsis

Most of the analyzed Thraustochytriaceae were located in the first major branch of the phylogenetic tree.

Eleven strains belonged to the genus *Ulkenia sensu stricto*, as described by Yokoyama et al. [64]. They were divided into two main branches, as also described elsewhere [62,64,87,89,90,91,99]. One contained strains closely related to *U. profunda*, and the other contained strains near *U. visurgensis*. Carotenoid cluster analysis revealed three main clusters containing *Ulkenia* spp. with high similarity to the phylogenetic tree. One of them contained the strains belonging to *U. profunda*, while the other two contained strains related to *U. visurgensis*. Morphology showed some unique properties of this genus, such as “hatching” and perfectly globose cells. Three strains belonging to *Ulkenia* spp. showed a significant need for higher salinities for growth in the DoE experiments, which was especially pronounced in two strains belonging to *U. profunda* (N5905 and N5629e).

*Thraustochytrium* is a polyphyletic genus and contained most (33) of the analyzed strains in four different clades, belonging to the species *T. kinnei*, *aureum*, *striatum*, and *aggregatum*. Although they were distributed across the phylogenetic tree, the general placement of each clade was consistent with literature data.

Strains related to *T. kinnei* synthesized medium amounts of squalene but no carotenoids. Their morphology was most often unobtrusive with the special characteristics of oblong, branched cellular substructures under certain conditions. *T. kinnei* was most closely related to *S. aggregatum* in this phylogeny and elsewhere [87,89,90,91]. Strains related to *S. aggregatum* resembled *T. kinnei* only in that carotenoid production was low and squalene content was intermediate. They differed in that *S. aggregatum* had higher biomass yields. They were quite distinct from the other clades based on 18S rRNA phylogeny and carotenoid cluster analysis.

Most strains in the clade of *T. aureum* excelled by high biomass yield, high carotenoid content, but very low squalene content. Possibly, they are also potent fatty acid producers. *T. striatum* N5997 was the closest relative to strains of this clade. The closest neighbors (based on 18S rRNA genes) to *T. striatum* were the clades of *T. aureum*, *Thraustochytriidae* sp., and *Ulkenia.* Similar relationships were also reported by other authors [65,78,81,87,90]. *T. striatum* N5997 also showed a comparably high biomass yield and carotenoid content. In contrast to *T. aureum*, it exhibited elevated squalene levels. However, its carotenoid composition was more similar to strains belonging to *Ulkenia*, which was distantly related. Like in *Ulkenia* spp., the phenomenon of “hatching” cells was observed in *T. striatum*. Due to their high biomass and carotenoid yields, *T. aureum* and *T. striatum* might be interesting for further investigation. *T. aggregatum* was quite isolated in the phylogeny, which was also displayed by other researchers [63,64,78,87,88,89]. It synthesized no carotenoids but was characterized by a rapid color change from white to red at varying cultivation conditions and small and well-dispersed cells in the morphological observations. Therefore, its distant position in the 18S rRNA sequence-based phylogeny was reinforced by its other characteristics.

Three strains assigned to *Thraustochytriidae* sp. were in the first instance related to *Labyrinthulochytrium* spp. and more distantly related to *Phycophthorum* sp. and *Ulkenia* spp. They were also closely related to *Thraustochytriidae* sp. M4-103*, which was shown to be related to *L. haliotidis* and *Ulkenia* spp. [88]. However, a closer proximity of *L. haliotidis* to *T. kinnei* than to *U. profunda* was also described [68,88,182]. The carotenoid patterns of strains related to *Thraustochytriidae* sp. were shown to be similar to those of members of *T. aureum*, although their phylogenetic relationships were quite distant.

Strains classified as *O. minutum* were located in the second major branch of the dendrogram. A clear phylogenetic distinction from most of the other members of Thraustochytriaceae has been reported quite often [63,64,65,78,87,88,89,90]. No carotenoids were measured in these strains, but the production of substances that were assigned to porphyrins was observed. *O. minutum* was rearranged in 2007, formerly known as *Schizochytrium minutum* [63]. Based on the recorded characteristics, it was very different from the strains assigned to *S. aggregatum*, which produced small amounts of carotenoids, differed in morphology, and were located in the other major branch of the phylogenetic tree.

## 4. Materials and Methods

### 4.1. Chemicals and Reagents

Analytical grade acetone (SupraSolv) and acetonitrile (hypergrade) were obtained from Merck (Darmstadt, Germany). Ethanol and tris(hydroxymethyl)aminomethane (TRIS) (≥99.9%) were provided by Carl Roth (Karlsruhe, Germany), and formic acid (99% ULC/MS) by Biosolve (Valkenswaard, Netherlands). Standards of all-*E*-astaxanthin (SML0982, ≥97%), all-*E*-canthaxanthin (PHR1239, 96.7%), all-*E*-lutein (PHR1699, 85.6%), all-*E-β*-carotene (PHR1239, 97.6%), and squalene (442785, analytical standard) were provided by Sigma-Aldrich (Taufkirchen, Germany). All-*E*-zeaxanthin (10009992, ≥98%) was provided by Cayman Chemical (Ann Arbor, MI, USA). All-*E*-lycopene (0031, ≥95%), all-*E*-echinenone (0283, ≥95%), 9*Z*-canthaxanthin (0380.1, ≥95%), all-*E*-phoenicoxanthin (0391), all-*E*-antheraxanthin (0231, ≥95%), all-*E*-violaxanthin (0259, ≥95%), all-*E*-rhodoxanthin (0424, ≥95%), and astaxanthin monopalmitate (1017, ≥95%) were obtained from CaroteNature (Münsingen, Switzerland).

Premium Sea Salt was obtained from Dupla Marin (Grafschaft-Gelsdorf, Germany), yeast extract, meat peptone, agar, NaHCO_3_, MnCl_2_ x 4 H_2_O, cobalamin, biotin, and thiamin hydrochloride were provided by Carl Roth (Karlsruhe, Germany). MgSO_4_ × 7 H_2_O, KH_2_PO_4,_ FeCl_3_ × 6 H_2_O 9 mg/L, ZnSO_4_ × 7 H_2_O, CoSO_4_ × 7 H_2_O, CuSO_4_ × 5 H_2_O, NaOH (≥98%), and HCl (37%) were obtained from Merck (Darmstadt, Germany), and riboflavin from AppliChem (Darmstadt, Germany).

### 4.2. Culture Collection

The 60 strains used were all part of the former “Kulturensammlung mariner Pilze Bremerhaven” (KMPB) of the Alfred-Wegener-Institut für Polar und Meeresforschung (Bremerhaven, Germany). It was originally established in the 1970s and contains strains, some of which have already been described [61,62,88,92,105,168,183,184,185,186]. This collection is now part of the Flensburg strain collection of marine fungi (mFSC). The following strains were used: 154f*, 561bx*, 1450d, 1462d*, 1465d*, 1471d, 1471f, 1472e, 1473e, 1476b, 1485a, 1518e, 1526d, 1527a, 1527c, 1531c, 3041c*, 4992b*, 5985*, 5996*, 5999*, N557a, N561b, N1001, N1438e*, N1476c*, N1694d* (accession number: L34668), N1709d*, N2820a*, N2845c, N4930a, N4930b, N4994b, N4994d*, N4995d*, N5589c*, N5594d*, N5629e*, N5658a*, N5661, N5670c*, N5676f, N5905*, N5976*, N5995*, N5997*, N5998*, N6000b*, N6001b, N6002a, N6005a, N6006d*, N6006e, N6007e*, N6421, N6422, N6423, N6424, N6523, and Sakar7*. The asterisks indicate the 30 strains used for 18S rRNA gene analysis, growth and terpene studies. All other strains were analyzed only regarding their 18S rRNA genes. Some strains were analyzed in earlier works [187,188].

### 4.3. Culture Media, Cultivation, and Harvest

The following media were used for the maintenance of the cultures: B1TM, B1TMG and AS. The basis of B1TM was adapted according to Gupta et al. [189] and included sea salt 30 g/L, yeast extract 1 g/L, meat peptone 1 g/L, trace element solution 1 mL/L and vitamin mix 1 mL/L. B1TMG included sea salt 15 g/L, yeast extract 5 g/L, meat peptone 5 g/L, glucose 10 g/L, trace element solution 1 mL/L, and vitamin mix 1 mL/L. The AS medium included sea salt 28.5 g/L, glucose 1 g/L, casein peptone 1.8 g/L, trace element solution 1 mL/L and vitamin mix 1 mL/L. Its pH was adjusted to 6.9 with 1 M HCl before sterilization. Media for the design of experiments (see below) were prepared likewise but with agar 12 g/L. Their pH was adjusted with 1 M NaOH or 1 M HCl.

The trace element mix was adapted from Lee Chang et al. [190]: MgSO_4_ × 7 H_2_O 200 mg/L, KH_2_PO_4_ 200 mg/L, NaHCO_3_ 100 mg/L, MnCl_2_ × 4 H_2_O 9 mg/L, FeCl_3_ × 6 H_2_O 9 mg/L, ZnSO_4_ × 7 H_2_O 1 mg/L, CoSO_4_ × 7 H_2_O 0.34 mg/L, and CuSO_4_ × 5 H_2_O 0.2 mg/L. It was prepared by autoclaving a stock solution of each component individually and combining them under sterile conditions to the final concentration. The vitamin mix was prepared based on Bahnweg [92] for a final concentration in the medium of: cobalamin 5 µg/L, biotin 10 µg/L, thiamin hydrochloride 10 µg/L, and riboflavin 10 µg/L. All vitamins were prepared individually in stock solutions. Filter sterilized (0.2 µm) stocks were combined and diluted to their final concentrations with sterile millipore water. Trace elements and vitamins were added sterilely to the culture media after autoclaving and cooling.

The used strains were defrosted from cryopreservation (−80 °C) and suspended in either 20 mL B1TM, B1TMG or AS liquid medium, and brought into culture in Erlenmeyer flasks. They were incubated at 20 °C and 70 rpm for 7–14 days in the dark. If the growth was sufficient, i.e., the medium had become turbid or visible pellets had been formed, the first passage was performed after seven days. Otherwise, the cultures were cultivated under the same conditions until observable growth. The inoculation volume for each passage was 1 mL. The cultures were passaged every 7–9 days until the culture was either harvested for DNA analysis or used to inoculate the design of experiment (DoE) media and morphology experiments. For DNA analysis, either an aliquot of 1 mL was taken from the culture and transferred to a reaction tube, or the whole broth was transferred to a 20 mL Falcon tube and centrifuged at 1000× *g* for 5 min. Subsequently, most of the supernatant was removed; the cell pellet was resuspended and transferred to a reaction tube. Storage until the next steps was at −21 °C.

For inoculation of the DoE, 400 µL of the 7–9 day old culture was transferred on each petri dish containing one of the described DoE media (see below). The same medium used for the liquid cultures served as a positive in form of solid agar. B1TMG was used as positive control for N6006d, 561bx, 154f, N4995d, N2820a, N4994d, N6007e, N1694d, N5998, N1709d, 1465d, N1476c, 4992b, 5985, 5999, N5658a, N5997, N5594d, 3041c, N5589c, N5976, N5670c, and Sakar7. B1TM was used for N5995, N5629e, N5905 and AS was used for 5996 and N6000b. Cultures were incubated for 13 days in the dark at 20 °C. Subsequent harvesting was performed by scraping the biomass with a spatula and weighing directly into lysis tubes type C (Macherey Nagel, Düren, Germany). The biomass was deep-frozen at −21 °C until further processing.

### 4.4. 18S rRNA Gene Characterization and Phylogeny

In a first step, DNA was extracted from the harvested cell pellet. Therefore, the Quick-DNA Microprep Kit (Zymo Research, Freiburg, Germany) was used, and an adapted version of the protocol “solid tissue samples” was followed. First, 100–150 µL biomass was added to 500 µL Genomic Lysis Buffer from the kit in a lysis reaction tube type C (Analytik Jena, Jena, Germany). Cell disruption was performed using a vibration mill (Retsch GmbH, Haan, Germany) at a frequency of 27 Hz and a duration of 2 × 3 min with a break of 30 s in between. The lysate was centrifuged at 10,000× *g* for 5 min. Further steps were executed according to the manufacturer’s protocol.

The PCR reaction mixture consisted of the following components: 25 µL DreamTaq Green PCR Master Mix (Thermo Scientific, Waltham, MA, USA), 23 µL nuclease-free water (Carl Roth, Karlsruhe, Germany), 1 µL DNA template, 0.5 µL forward primer (100 µM) and 0.5 µL reverse primer (100 µM). For PCR, primers T18S1F and T18S5R [95] were used. The amplification was performed similarly as described by Burja et al. [95], using a Mastercycler gradient thermocycler (Eppendorf, Hamburg, Germany). Initial denaturation was at 95 °C for 180 s. Thirty cycles were performed of 30 s at 95 °C, 30 s at 56 °C (annealing), and 90 s at 72 °C. Final extension was conducted at 72 °C for 10 min. The PCR products were checked by gel electrophoresis, and well-amplified sequences were sequenced by Sanger sequencing. Non-sufficient PCR products were discarded, and the original DNA was again amplified with primers F and RA2 [191] with the identical PCR program and FA1 [191] and SR-11 [192] with an adapted annealing temperature at 49 °C. Sanger sequencing was carried out at the Institute for Clinical Molecular Biology (IKMB) at Kiel University. In addition to the primers used for PCR, the following primers were used for sequencing: FA2, FA3, R, RA1, RA3 [191], and SR-6 [192].

The sequences were assembled with ChromasPro (version 2.1.9, Technelysium Pty Ltd., South Brisbane, Australia). In addition to these sequencing data, 18S rRNA gene sequences from closely related species found by a BLAST search, further members of the families of the Thraustochytriaceae and Labyrinthulaceae and strains used as outgroup* were retrieved from the GenBank database. They were added to the alignment and the phylogenetic tree to increase robustness and informative value, and to enable a well-founded and meaningful discussion (NCBI accession numbers AB022103.1, AB022104.1, AB022106.1, AB022108.1, AB022109.1, AB022110.1, AB022111.1, AB022112.1, AB022113.1, AB022116.1, AB126669.1, AB290355.1, AB290575.1, AB290576.1, AB290577.1, AB355410.1, AB355411.1, AB355412.1, AB636147.1, AB810962.1, AB810968.1, AB810969.1, AB810977.1, AB973514.1, AB973517.1, AB973524.1, AB973531.1, AB973545.1, AB973546.1, AB973561.1, AB973564.1, AB973565.1, AJ415519.1*, AJ519935.1, AY705753.2, DQ023615.1, DQ367049.1, DQ367050.1, DQ374149.1, EF114348.1, FJ004948.1, FJ821482.1, FJ799799.1, FR875335.2, GQ452862.1*, HQ228958.1, HQ228964.1, HQ228969.1, HQ228980.1, JQ281514.1*, JX993839.1, JX993841.1, KF500513.1, KT598545.1, KT598546.1, KT716334.1, KX160007.1, KX379459.1, KX430103.1, L34054.1, L34668.1, MF872140.1, MG099001.1, MG799152.1*, MK615597.1, MN382127.1, MT484273.1, and U21338.1). All successfully assembled sequences that belonged to the Thraustochytriaceae were aligned using the Clustal W algorithm. The phylogenetic tree was based on the maximum-likelihood algorithm and the nucleotide substitution model by Tamura and Nei [193]. Missing gaps were treated by partial deletion, i.e., positions with less than 95% site coverage were eliminated. The final dataset contained 86 sequences and 1378 base positions. The phylogeny was tested using the Bootstrap method with 1000 replicates and rooted using the outgroup. Alignment and tree building were performed with MEGA 11 (Molecular Evolutionary Genetics Analysis version 11, Tamura, Stecher, and Kumar 2021 [194]). All obtained sequences were deposited in the NCBI GenBank database under accession numbers given in Table S1.

### 4.5. Design of Experiment and Model Regression—Growth Studies

A two-step approach was used to determine the optimal growth parameters. First, a series of different media was created using a statistical experimental design. The recipes were prepared as solid media, inoculated, and incubated with different strains. Different models were calculated based on the biomass yield and compared. The most applicable model was used to evaluate the growth behavior of the different strains.

For the design of experiments, four variables were chosen: Glucose (0–60 g/L), yeast extract (0.5–15 g/L), pH (6.5–7.6), and additional potassium dihydrogen phosphate (0–0.5 g/L) to a base concentration of approximately 0.06 mg/L. In an additional set of experiments, which was used only for a smaller number of strains, the salt concentration (15–30 g/L) was used as a further variable (Table 4). Constant parameters were temperature (20 °C), trace element solution (1 mL/L), vitamin mix (1 mL/L), agar (12 g/L), and, for the first set of experiments, salt concentration (15 g/L). It was expected that some strains were oligotroph, so the design was established in two subsets: For better coverage of the area with lower concentrated media components, the first subset of 6 media was calculated with reduced media components, using a Latin hypercube algorithm and space-filling design. The second subset of six additional media was created with the above concentrations and a space-filling design (all JMP PRO, version 16.0.0, SAS Institute Inc., Cary, NC, USA). Additionally, three runs with nearly extreme (high or low) amounts of glucose and yeast were added (Table 4 No. 13, 14, 15). As a response, the biomass yield was used.

The obtained results were regressed using the standard least squares method with different models (JMP PRO). All experiments executed without further addition of salt were regressed by the following two approaches: quadratic (Equation (1)), reduced quadratic including one interaction term (glucose and yeast extract) (Equation (2)), and reduced quadratic including the interaction terms of glucose and yeast, glucose and pH, glucose and phosphate, yeast extract and pH, and yeast extract and phosphate (Equation (3)). *Y* is the yield, *X_1_* represents the glucose concentration, *X_2_* the yeast extract concentration, *X_3_* the pH, and *X_4_* the phosphate concentration. *a* is the intercept, *b_i_, c_i_,* and *d_ij_* are model coefficients.

(1) $$Y=a+\overset{4}{\underset{i=1}{\sum }}b_{i}X_{i}+\overset{4}{\underset{i=1}{\sum }}c_{i}X_{i}^{2}$$

(2) $$Y=a+\overset{4}{\underset{i=1}{\sum }}b_{i}X_{i}+\overset{2}{\underset{i=1}{\sum }}c_{i}X_{i}^{2}+d_{12}X_{1}X_{2}$$

(3) $$Y=a+\overset{4}{\underset{i=1}{\sum }}b_{i}X_{i}+\overset{2}{\underset{i=1}{\sum }}c_{i}X_{i}^{2}+d_{12}X_{1}X_{2}+d_{13}X_{1}X_{3}+d_{14}X_{1}X_{4}+d_{23}X_{2}X_{3}+d_{24}X_{2}X_{4}$$

The adjusted coefficients of determination were used to compare the different models. T-tests were applied to identify the statistical significance of the model parameters. The generally best describing-model was model 2. It was chosen for deeper investigation, and the influence of the parameters on yield was tested for significance. The gradient descent algorithm was used to numerically calculate the optimal medium composition. Different strains were compared by their predicted optimized yield. The influence of the salt concentration (regressor *X_5_*) on the growth of the strains was analyzed by the extension of model 2 by the linear influence of the salt concentration (Equation (4)) and by direct comparison of the growth on DoE media 6, 8, 13, 14, 15 with a low (15 g/L) and high (30 g/L) salt concentration.

(4) $$Y=a+\overset{5}{\underset{i=1}{\sum }}b_{i}X_{i}+\overset{2}{\underset{i=1}{\sum }}c_{i}X_{i}^{2}+d_{12}X_{1}X_{2}$$

### 4.6. Extraction and UHPLC Analysis of Carotenoids and Squalene

The harvested biomass was disrupted in acetone to extract carotenoids and squalene. Therefore, up to 1.5 g of wet biomass was put into lysis tubes type C (Analytik Jena, Jena, Germany or Macherey Nagel, Düren, Germany). An amount of 500 µL acetone was added, and the cells were broken mechanically in a vibration mill (MM 2000, Retsch, Haan, Germany) at 27 Hz for 3 min. After subsequent centrifugation at 10,000× *g*, the supernatant of the samples was transferred to a centrifuge tube, and 500 µL of fresh acetone was added to the lysis tube. This procedure was repeated until the supernatant and the residual biomass were colorless, but at least three times. The solvent of the combined supernatant was evaporated under a gentle stream of nitrogen at 40 °C. The remaining extract was dissolved in 250 to 500 µL acetone and filtered (0.45 µm, PTFE) into amber vials.

Qualification and quantification of carotenoids and squalene were performed by UHPLC using an ACQUITY Arc system by Waters (Milford, MA, USA) equipped with a sample manager (FTN-R), a quaternary solvent manager (R), an UV/Vis detector (2998 PDA Detector), and a mass spectrometer (Acquity QDa Detector). A C18-column (Cortecs C18 2.7 µm, 90 Å, 3.0 × 100 mm, Waters, Milford, MA, USA) was operated at 40 °C. The injection volume was 5 µL. The starting conditions were 70% millipore water (A) and 30% acetonitrile (B), containing 0.1% formic acid. Within 4 min, the gradient increased linearly to 90%B:10%A. This ratio was held isocratically for 5 min. Then B was increased linearly to 100% over 2.5 min and held for another 16.5 min. After a total of 28 min, the initial ratio was restored for 4 min to regenerate starting conditions. Flow velocity was 0.5 mL/min. Samples with a high extract content sometimes required additional cleaning of the column by running 100% acetonitrile at 60 °C for at least 30 min. Optical spectra were measured in a range of 200 to 800 nm, and carotenoid and squalene peaks were compared to the retention times of the standards. Data were analyzed and quantified at their determined absorbance maxima at 200 nm (squalene), 441 nm (violaxanthin), 448 nm (lutein and antheraxanthin), 452 nm (*β*-carotene), 454 nm (zeaxanthin), 461 nm (echinenone), 466 nm (9*Z*-canthaxanthin), 471 nm (lycopene), 475 nm (canthaxanthin), 477 nm (phoenicoxanthin and astaxanthin monopalmitate), 479 nm (astaxanthin), and 488 nm (rhodoxanthin). The mass spectrometer with electrospray ionization (ESI) was operated in positive mode with a cone voltage of 15 V and a probe temperature of 600°C, measuring in a range of *m*/*z* 150 to 1250. For further accuracy, the mass of selected carotenoids was observed by selected ion recording (SIR) at *m*/*z* 537.4 [M+H]^+^ (*β*-carotene), 551.4 *m*/*z* [M+H]^+^ (echinenone), *m*/*z* 565.4 [M+H]^+^ (canthaxanthin), and *m*/*z* 597.4 [M+H]^+^ (astaxanthin). Besides the major all-*E*-astaxanthin peak, peaks with UV/Vis absorption spectra corresponding to *Z*-astaxanthin isomers [195,196,197,198,199,200] that were additionally accompanied by peaks with the mass of astaxanthin in SIR were assigned to astaxanthin diastereomers. Their quantities were estimated using the quantification of all-*E*-astaxanthin, corrected by factors adjusting the different extinction coefficients published by Bjerkeng et al. [201], namely 1.20 for 9*Z*-astaxanthin, 1.56 for 13*Z*-astaxanthin, and 1.11 for the di-*Z*-isomers. Astaxanthin monopalmitate was detected by comparison of spectra and retention time with a standard. The carotenoid part was quantified using the calibration curve for all-*E*-astaxanthin. If no further information is given, the name of the carotenoid always stands for its all-*E*-isomer.

For identification and quantification, standards were used at concentrations of 0.2–50.0 µg/mL in acetone. Calibration curves were adjusted to the concentration range of the substance in the samples. Blank acetone was applied for zero-value determination. Linear regression of the calibration data was performed by the ordinary least squares method, and the calibration was forced through zero. The content of unknown carotenoids was estimated by a mean calibration of all analyzed carotenoid standards. Dereplication was performed by comparison of retention times, UV/VIS absorption-, and mass spectra.

### 4.7. Target Molecule Content, Composition, Cluster- and Regression Analysis

The ability of the strains to produce carotenoids and squalene was evaluated using samples from the growth studies. 1–4 plates per strain were selected based on the strongest coloration and the highest biomass for a detailed comparison. These were harvested separately from the others, and the biomass was directly transferred into lysis tubes after weighing. The biomass of all other plates was collected into one lysis tube after weighing. Samples were extracted and measured. For the calculation of target molecule content on a dry weight basis, a generalized cell dry weight (CDW) was assumed. It was determined in the experiments for the effect of medium composition (see below). The mean content of the target molecules was calculated using the harmonic mean. Cluster analysis of the obtained patterns was performed using the k-means algorithm and hierarchical clustering (JMP PRO). Due to possible differences in scale and variability, datasets were standardized for both approaches. For strain *T. striatum* N5997, all 15 plates were harvested and analyzed separately to regress squalene and carotenoid content (µg/mg) and yield (µg/L) on various growth parameters. To increase explanatory power, model 2 of the growth regression was extended if necessary. In this manner, models specifically adjusted to describe the growth and target molecule production of this specific strain were developed.

### 4.8. Morphology

Strains from different clades in the created phylogenic tree were used for morphological analysis (Sakar7, N6000b; N5994d, N5589c N5629e, N5905, N5658a; N5670c, N4994d; N6007e, N6006d; 561bx, N2820a, 5999; N147c, N1694d; N4930a, 4992b; N5997; 5996, N5995). To record their physiological appearance under different cultivation conditions (different amount of yeast extract, glucose, phosphate, and different pH), the experiment was performed in four liquid media derived from the DoE (number 3, 6, 12, and 14). B1TMG was used as a positive control. The experiment was carried out in 12 well microtiter plates with 2.5 mL medium per well. The inoculation was performed with 80 µL from 7-day-old liquid stock cultures. To allow uniform air diffusion, the wells in the center of the plates functioned as blanks. The plates were incubated for 13 days in the dark at 20 °C without shaking. Subsequently, the first step was a microscopic evaluation using an inverted microscope (Eclipse TS100, Nikon, Tokyo, Japan). The plates remained sealed, and no samples were taken, so the reticular structures remained undamaged. In the next step, a conventional microscopic assessment (Carl Zeiss Microscopy GmbH, Jena, Germany) was carried out. Various criteria were considered for evaluating the morphology: cell size, cell form, agglomeration behavior, formation and properties of ectoplasmic nets (EN), the appearance of sporangia and spores, and extracellular and intracellular structures.

### 4.9. Effect of Medium Composition on the Target Molecules

DoE experiments 6, 9, and 15 were repeated with selected strains (N5670c, N4994d, N6000b, N1001, N5589c, N5594d, N5658a, 5996, N2820a, 5999, N6006d, 5985) to quantify the target molecules on a CDW basis. They were performed similarly to the DoE but in multiple executions for a higher biomass yield. The biomass was harvested, measured, filled into micro-reaction tubes, and frozen at −80 °C before freeze-drying (Alpha 1–4, Christ, Osterode, Germany) for 24 h at 37 Pa. The weight of the biomass was recorded before and after drying, and CDW was calculated. Extraction and measurement of the target molecules were performed as described above.

### 4.10. Comparison of Biomass Yield and Target Molecule Content in Cultures Cultivated in Liquid and on Solid Medium

To compare the biomass growth and carotenoid synthesis, selected strains (N6006d, N4994d, N5658a, N1001) were cultivated on medium 7 from the DoE. It was either prepared with 12, 14, and 18 g/L agar or without agar. Inoculation was performed similarly to the previous experiments from the liquid stock cultures. Liquid cultures inoculated with 1 mL from the stock culture were cultivated in Erlenmeyer flasks with 20 mL medium on an orbital shaker at 80 rpm. Solid and liquid cultures were incubated for 13 days in the dark and at 20 °C. Harvest of the cultures from the solid media was performed as described for the DoE. As no color difference between the solid media was visible, these were combined. The cultures from the flasks were transferred into centrifuge tubes and centrifuged for 5 min at 4000× *g*. The supernatant was discarded and replaced with distilled water. The sample was resuspended by vortexing for 30–60 s. Centrifugation and washing steps were repeated twice. Afterward, the supernatant was discarded, the cell pellet was resuspended in 0.5 mL distilled water, transferred in glass tubes, and frozen at −80 °C prior to freeze-drying (Alpha 1–4, Christ, Osterode, Germany) for 24 h at 37 Pa.

## 5. Conclusions

The family of Thraustochytriaceae is very heterogeneous in its characteristics and traits. Although members possess many common features in their morphology, growth behavior, and metabolite patterns, these are not necessarily correlated to the phylogenetic clades based on 18S rRNA analysis. Within the described clades, some strains that were identical based on their 18S rRNA genes exhibited different characteristics. The carotenoid composition of *T. aureum* N5998 differed substantially from those of the other strains of this clade, although its 18S rRNA was genetically identical to two of them. We agree with the statement of Dellero et al. [125] and argue that gene-based analysis, especially using the short 18S rRNA information, is not able to predict the exact behavior and potential of individual strains.

A deeper understanding of the relation of these organisms might be achieved mainly by two aspects: First, there needs to be an enhanced awareness of the variability of this group, and there is a necessity to precisely classify existing genera and species to allow coherent comparison. 18S rRNA analysis is a useful tool for a general classification in terms of family and genus. A rearrangement, as performed for species formerly belonging to *Ulkenia*, *Schizochytrium*, and *Thraustochytrium* [63,64,202], might also be helpful for other clades of the polyphyletic genus *Thraustochytrium* to allow better distinction. Second, the variety of Thraustochytriaceae is still underexplored. Recent discoveries of new genera, species [88,96,110,125,181,203], and strains, especially members of the genus *Aurantiochytrium,* were described [78,89,99,120,204]. Additionally, the high number and variability of novel isolates displayed in the phylogenetic tree published by Ueda et al. [91] indicates that the Thraustochytriaceae may contain more species and genera, which either have new characteristics or bridge the gaps between others. A comprehensive and correct denomination of novel species is crucial for a distinguished and meaningful discussion.

18S rRNA phylogenetic analysis allowed the distinction of the strains and assigned them to eight different clades. Carotenoid cluster analysis revealed that a fine classification at a species level was possible but failed to reflect the overall context of the phylogenetic tree based on 18S rRNA gene comparison. Strains belonging to *T. aureum* and *Thraustochytriidae* sp. showed the highest carotenoid content. Most strains of *T. aureum* also yielded high biomass. Squalene was found in variable concentrations in all strains. The conditions that favored carotenoid and squalene production were largely mutually exclusive. *T. striatum* N5997 produced squalene and carotenoids at intermediate levels and built high biomass, which might be a good compromise for a combined production.

The results of these experiments indicate that Thraustochytriaceae offer more potential for biotechnological applications than just the genus *Aurantiochytrium*. Closer examination of other clades for their ability to synthesize carotenoids, squalene, and fatty acids might help to find new production organisms. Subsequently, bioprocess, metabolic, and genetic engineering approaches will provide a basis for a sustainable production using the fascinating group of Thraustochytriaceae.

## Figures and Tables

**Table 1:** Estimated model coefficients, *p*-values, and optimized parameters of model 2 for four exemplary strains regarding maximal biomass yield. Significant *p*-values are indicated with ** (σ = 0.01) and * (σ = 0.05). The variance of the yield is given for a 0.95 confidence interval.

	*T. aureum* N6006d		*U. visurgensis* N5594d		*Thraustochytriidae* sp. N4994d		*T. kinnei* N1694d	
	Coefficient	*p*-Value	Coefficient	*p*-Value	Coefficient	*p*-Value	Coefficient	*p*-Value
a	−39.2		66.8		188.4		236.5	
b_1_ (G ^a^)	−0.4	0.0009 **	-0.01	0.0030 **	0.7	0.0014 **	0.09	<0.0001 **
b_2_ (Y ^b^)	69.0	0.0137 *	−4.4	0.00055 **	6.5	<0.0001 **	2.91	<0.0001 **
b_3_ (pH)	−6.5	0.9614	−4.4	0.6555	−24.8	0.0397 *	−28.9	0.0086 **
b_4_ (P ^c^)	246.7	0.5375	−13.4	0.5185	−30.0	0.1807	−66.2	0.0058 **
c_1_ (G²)	0.05	0.7913	0.01	0.5328	−0.01	0.2596	−0.01	0.5564
c_2_ (Y²)	−5.0	0.1615	0.5	0.0222 *	−0.3	0.2000	−0.1	0.4664
d_12_ (G*Y)	1.5	0.0200 *	0.03	0.02682	0.1	0.0060 *	0.16	0.0002 **
R²	0.9140		0.9322		0.9761		0.9533	
R² adj.	0.8281		0.8643		0.9482		0.9667	
*p*-value	0.0029		0.0013		0.0002		<0.0001	
Optimized parameters								
G	60		60		60		60	
Y	15		15		15		15	
pH	6.5		6.5		6.5		6.5	
P	0.5		0		0.5		0.5	
Y ^d^	25.9 ± 15.9		2.2 ± 0.6		2.8 ± 0.7		3.4 ± 0.6	

^a^ G, Glucose (g/L), ^b^ Y, Yeast extract (g/L), ^c^ P, KH_2_PO_4_ addition (g/L), ^d^ Y, Yield (g/L).

**Table 2:** Model quality and optimized parameters of models for growth, carotenoid, and squalene production in *T. striatum* N5997. Dry weight was approximated using data from the other trials.

		Target Molecule Content				Target Molecule Yield			
	Biomass Yield (g/L)	Squalene (mg/g)	Total Carotenoids (µg/g)	Total Astaxanthin (µg/g)	Phoenico- Xanthin (µg/g)	Squalene (mg/L)	Total Carotenoids (mg/L)	Total Astaxanthin (mg/L)	Phoenico-Xanthin (mg/L)
R²	0.9578	0.9887	0.8909	0.8956	0.9891	0.8530	0.9600	0.9622	0.9476
R² adj.	0.8819	0.9705	0.7164	0.7288	0.9644	0.5884	0.8601	0.8676	0.8532
*p*-value	0.0061	0.0002	0.0446	0.0403	0.0014	0.1052	0.0215	0.0194	0.0103
Optimized parameters									
G ^a^	60	0	48.7	48.4	60	29.9	60	60	60
Y ^b^	3.9	15	0.5	0.5	0.5	8.2	0.5	0.5	0.5
pH	7.6	7.6	7.6	7.6	7.6	7.6	7.6	7.6	7.6
P ^c^	0.5	0	0.5	0.5	0.5	0.3	0.5	0.5	0.5
Y ^d^	23.2 ± 7.5	34.8 ± 5.7	125.0 ± 47.4	120.1 ± 44.5	8.3 ± 1.1	40.0 ± 25.0	1.7 ± 0.6	1.5 ± 0.5	0.12 ± 0.04

^a^ G, Glucose (g/L), ^b^ Y, Yeast extract (g/L), ^c^ P, KH_2_PO_4_ addition (g/L), ^d^ Y, Yield.

**Table 3:** Retention times, UV/Vis absorption maxima, and masses of unknown substances found by UHPLC-PDA-MS chromatography in extracts of Thraustochytriaceae species. 1-4 carotenoids (assignment to strains and media in Tables S5–S8, S10, and S11), A–D porphyrins.

Substance	Retention Time (min)	λ_max_ (nm)	*m*/*z*	Species
Unknown 1	9.0	448	584.3 ± 1.1, 802.7 ± 0.2	*T. aureum*
Unknown 2	10.4	461	549.8 ± 0.7, 566.4 ± 1.2	*Ulkenia* spp., *Thraustochytriidae* sp., *T. aureum*
Unknown 3	14.8	462	n.d. ^1^	*Ulkenia* spp., *Thraustochytriidae* sp., *T. striatum*
Unknown 4	21.9	461, 489, 439sh	n.d.	*Ulkenia* spp., *Thraustochytriidae* sp., *T. striatum*, *S. aggregatum*
Unknown A	5.9	415, 548, 582	n.d.	*O. minutum* N5995
Unknown B	6.3	400, 502, 538	n.d.	*O. minutum* N5995
Unknown C	6.8	401, 504, 538, 574, 628	563.4 ± 0.02	*O. minutum* N5995
Unknown D	8.9	404, 508, 542	n.d.	*O. minutum* N5995

^1^ n.d. = not detectable.

**Table 4:** Overview of media composition of the experimental design for growth model establishment. Asterisks indicate media additionally prepared with a salt concentration of 30 g/L.

Number	Glucose(g/L)	Yeast Extract (g/L)	KH_2_PO_4_(g/L)	pH
1	10.0	2.3	0.50	6.9
2	2.0	1.4	0.00	7.3
3	0.0	3.2	0.40	7.6
4	4.0	5.0	0.21	6.5
5	6.0	0.5	0.30	7.5
6 *	8.0	4.1	0.10	7.2
7	27.5	14.8	0.05	6.7
8 *	58.8	12.9	0.08	7.6
9	55.4	6.1	0.46	7.4
10	32.9	11.7	0.21	7.0
11	16.4	11.0	0.44	7.4
12	46.8	8.8	0.32	7.0
13 *	0.5	0.5	0.20	7.2
14 *	0.5	15.0	0.28	7.0
15 *	60.0	0.5	0.15	7.3

## Data Availability

Data is contained within the article and supplementary material.

## Supplementary Materials

The following supporting information can be downloaded at: https://www.mdpi.com/article/10.3390/md21040204/s1, Figure S1: Carotenoid composition of the analyzed strains grown on media with a high salinity (30 g/L); Figure S2: Carotenoid composition of *Thraustochytrium striatum* N5997 on various DoE media; Figure S3: Morphology of various strains observed in medium 3 after cultivation in microtiter plates; Figure S4: Morphology of various strains observed in medium 6 after cultivation in microtiter plates; Figure S5: Morphology of various strains observed in medium 12 after cultivation in microtiter plates; Figure S6: Morphology of various strains observed in medium 14 after cultivation in microtiter plates; Table S1: Assignment of the investigated strains based on sequence similarity to strains in the NCBI database on the basis of their 18S rRNA gene data; Table S2: Coefficients of determination and *p*-values of model 1–3 for the regression of growth data; Table S3: Estimated model coefficients, *p*-values, and optimized parameters of model 2 for all analyzed strains regarding maximal biomass yield; Table S4: Biomass yield of the analyzed strains on the DoE media; Table S5: Carotenoid content and proportion in the analyzed strains used for cluster analysis; Table S6: Cluster means of carotenoid composition obtained from k-means clustering; Table S7: Carotenoid content and proportion in the analyzed strains in the DoE with a high salt concentration (30 g/L); Table S8: Carotenoid and squalene content and carotenoid proportion in *T. striatum* N5997 depending on the medium composition; Table S9: Extract content in dependency of strain, medium composition and medium solidity; Table S10: Carotenoid content and proportion in strains cultivated on different solid media; Table S11: Carotenoid content and proportion in strains cultivated on medium 7 in either liquid medium (liquid) or in form of agar (solid); Table S12: Review of strains reported in the literature by comparison with the phylogenetic analysis of our study.
