# Supplementary material for: Screening of a Thraustochytrid Strain Collection for Carotenoid and Squalene Production Characterized by Cluster Analysis, Comparison of 18S rRNA Gene Sequences, Growth Behavior, and Morphology
Source: Mar Drugs. 2023 Mar 24;21(4):204. doi: 10.3390/md21040204 (PMC10140983; doi:10.3390/md21040204)
Supplement: Supplementary file 1 [file marinedrugs-21-00204-s001.zip › SupplementFigures.pdf]

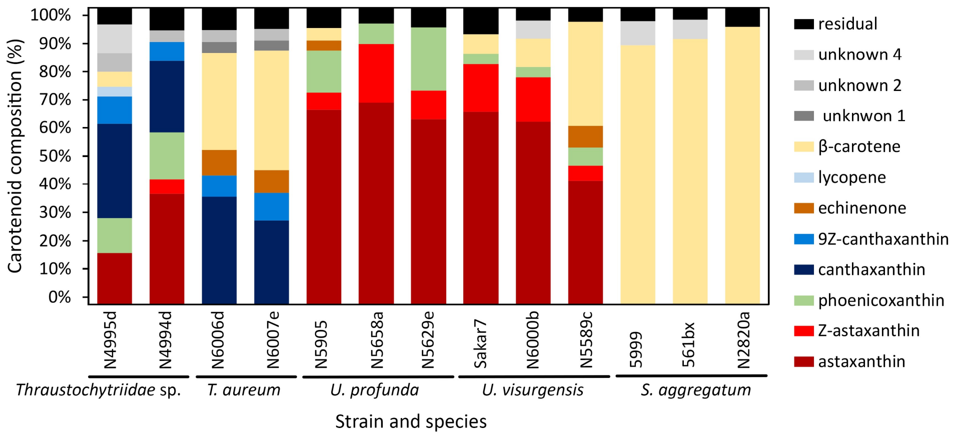

Figure S1. Carotenoid composition of the analyzed strains grown on media with a high salinity (30 g/L). Mean values of the individual experiments from each strain whose contribution to the total carotenoids exceeded 3% are displayed individually (Table S7). Diastereomers of astaxanthin are summarized as "*Z*-astaxanthin".

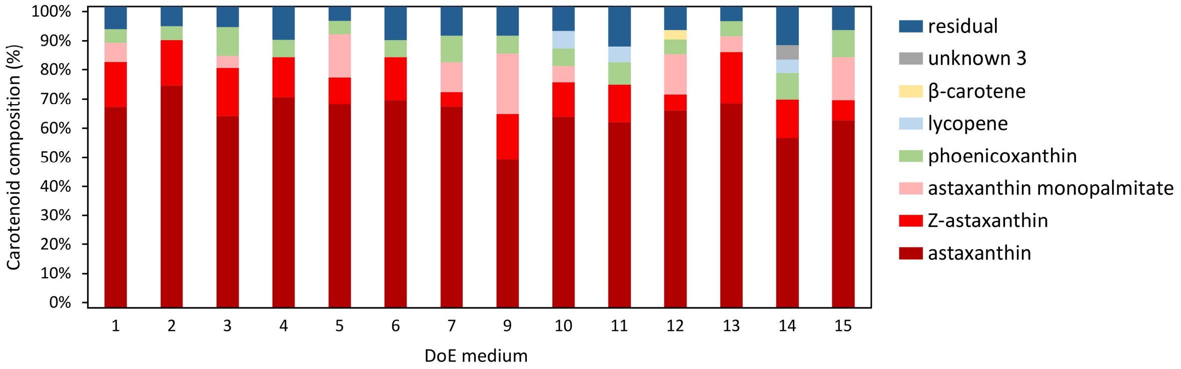

Figure S2. Carotenoid composition of *Thraustochytrium striatum* N5997 on various DoE media. Only carotenoids whose contribution to the total carotenoids exceeded 3% are displayed individually (Table S8). Diastereomers of astaxanthin are summarized as "*Z*-astaxanthin".

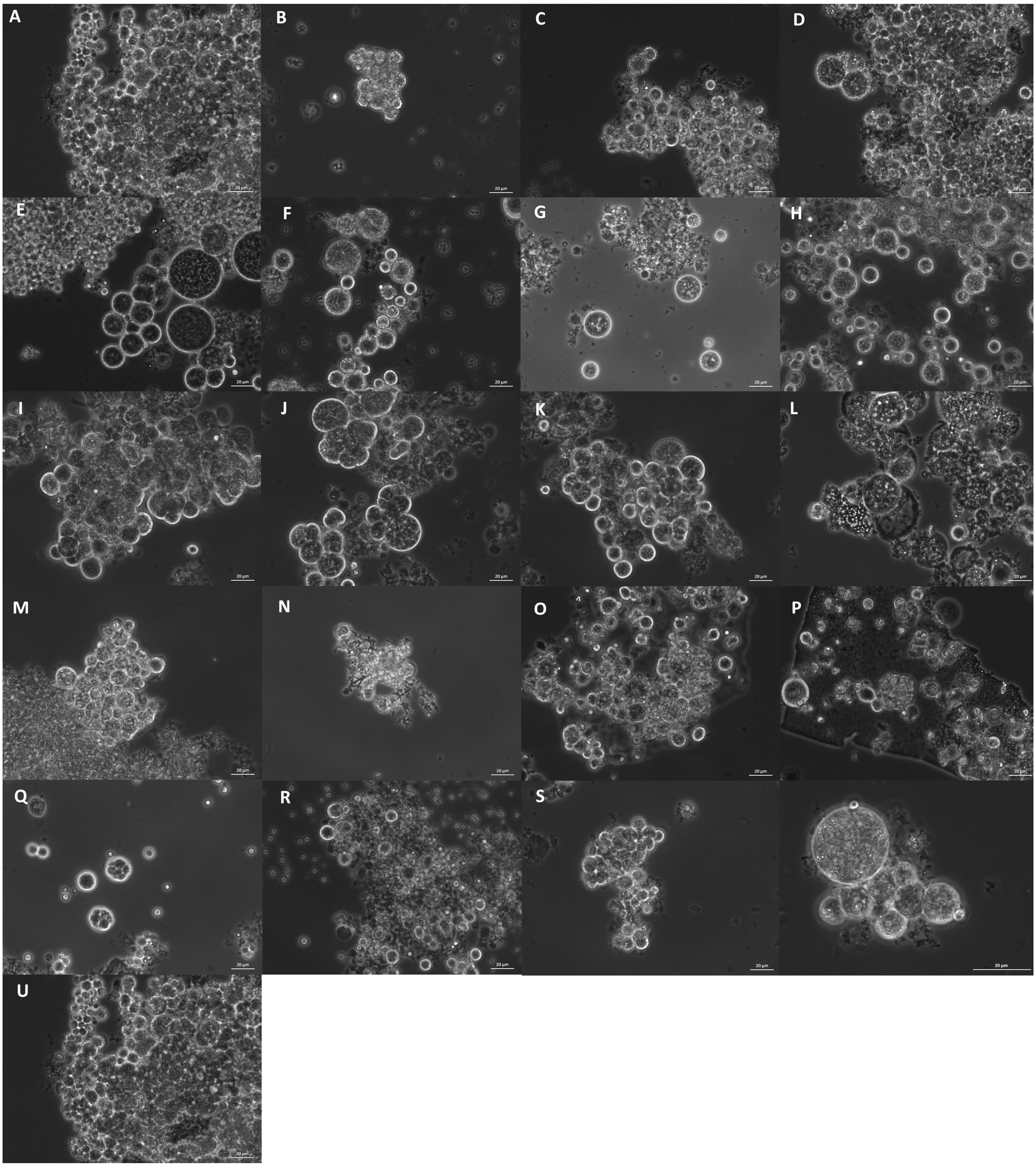

Figure S3. Morphology of various strains observed in medium 3 after cultivation in microtiter plates. *U. profunda* N5905 (A), N5658a (B), *U. visurgensis* 6000b (C), Sakar7 (D), N5594d (E), N5589c (F), *T. aggregatum* 4992b (G), N4930a (H), *S. aggregatum* 561bx (I), N2820a (J), 5999 (K), *T. striatum* 5997 (L), *O. minutum* N5995 (M), 5996 (N), *T. kinnei* N1694d (O), 1476c (P), *Thraustochytriidae* sp. N5670c (Q), N4994d (R), *T. aureum* N6006d (S), N6007e (T), and 5985 (U). Scale bars show 20 µm.

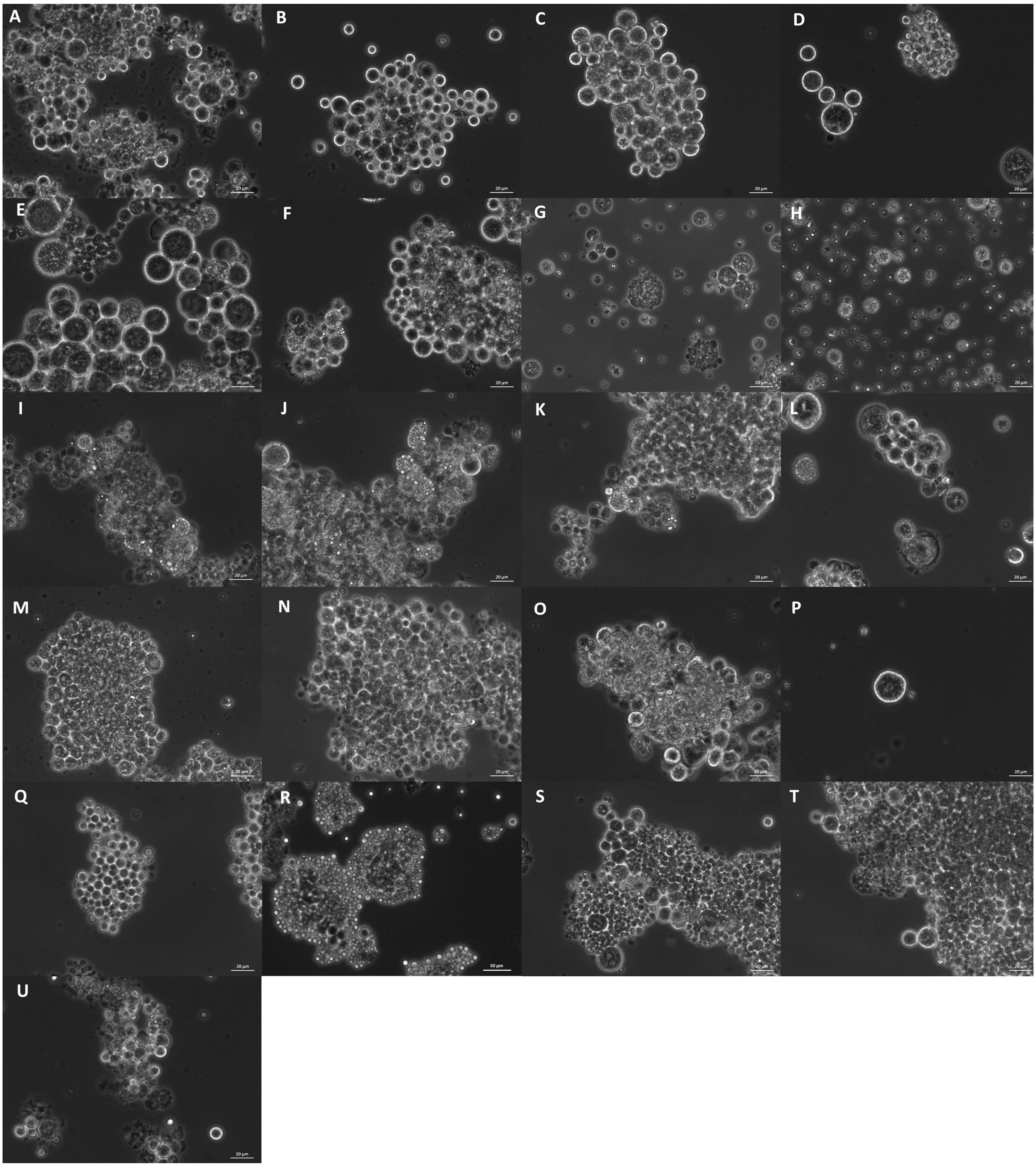

Figure S4. Morphology of various strains observed in medium 6 after cultivation in microtiter plates. *U. profunda* N5905 (A), N5658a (B), *U. visurgensis* 6000b (C), Sakar7 (D), N5594d (E), N5589c (F), *T. aggregatum* 4992b (G), N4930a (H), *S. aggregatum* 561bx (I), N2820a (J), 5999 (K), *T. striatum* 5997 (L), *O. minutum* N5995 (M), 5996 (N), *T. kinnei* N1694d (O), 1476c (P), *Thraustochytriidae* sp. N5670c (Q), N4994d (R), *T. aureum* N6006d (S), N6007e (T), and 5985 (U).

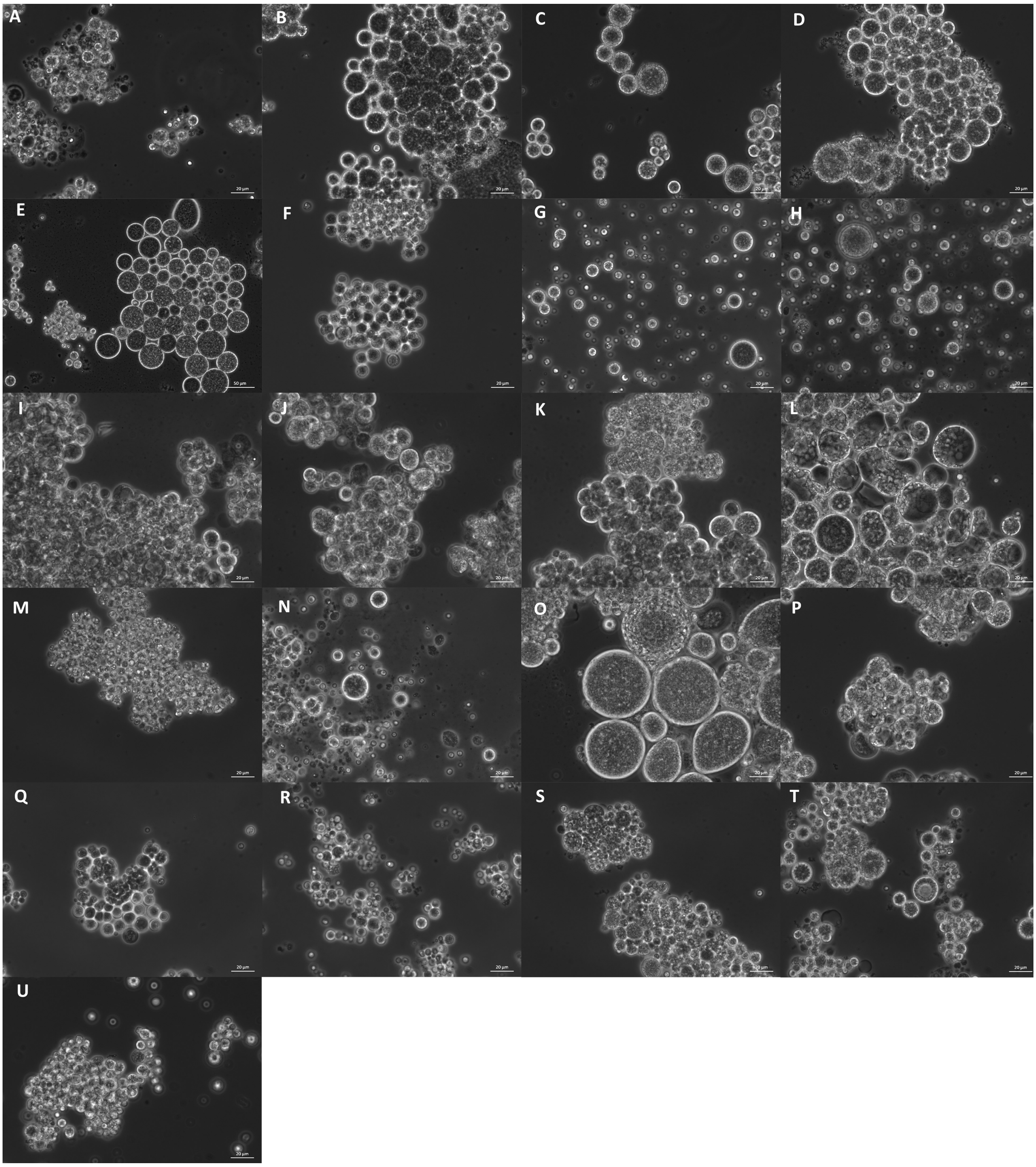

Figure S5. Morphology of various strains observed in medium 12 after cultivation in microtiter plates. *U. profunda* N5905 (A), N5658a (B), *U. visurgensis* 6000b (C), Sakar7 (D), N5594d (E), N5589c (F), *T. aggregatum* 4992b (G), N4930a (H), *S. aggregatum* 561bx (I), N2820a (J), 5999 (K), *T. striatum* 5997 (L), *O. minutum* N5995 (M), 5996 (N), *T. kinnei* N1694d (O), 1476c (P), *Thraustochytriidae* sp. N5670c (Q), N4994d (R), *T. aureum* N6006d (S), N6007e (T), and 5985 (U).

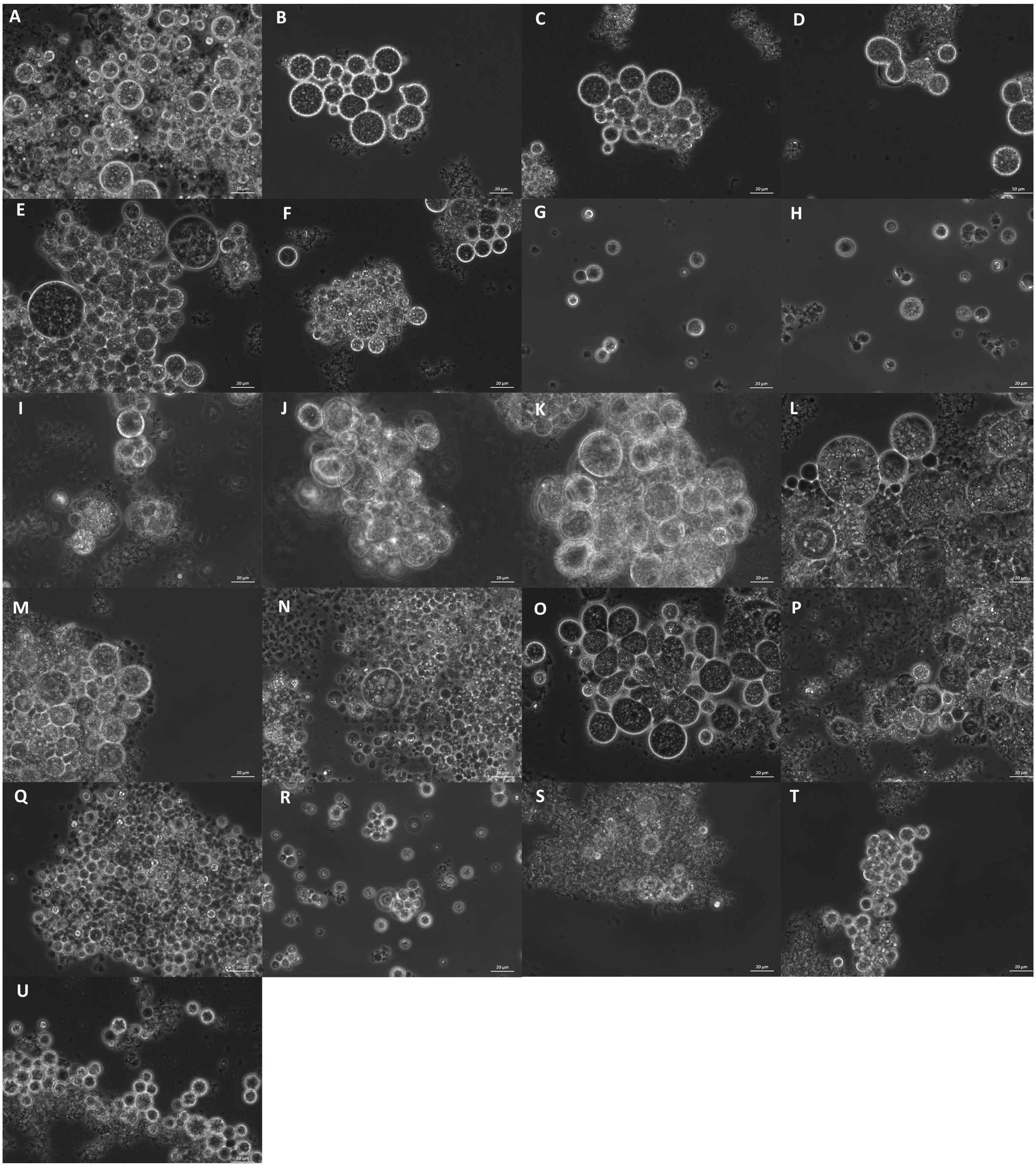

Figure S6. Morphology of various strains observed in medium 14 after cultivation in microtiter plates. *U. profunda* N5905 (A), N5658a (B), *U. visurgensis* 6000b (C), Sakar7 (D), N5594d (E), N5589c (F), *T. aggregatum* 4992b (G), N4930a (H), *S. aggregatum* 561bx (I), N2820a (J), 5999 (K), *T. striatum* 5997 (L), *O. minutum* N5995 (M), 5996 (N), *T. kinnei* N1694d (O), 1476c (P), *Thraustochytriidae* sp. N5670c (Q), N4994d (R), *T. aureum* N6006d (S), N6007e (T), and 5985 (U).
