# Supplementary figures and images for: Screening of a Thraustochytrid Strain Collection for Carotenoid and Squalene Production Characterized by Cluster Analysis, Comparison of 18S rRNA Gene Sequences, Growth Behavior, and Morphology
Source: Mar Drugs. 2023 Mar 24;21(4):204. doi: 10.3390/md21040204 (PMC10140983; doi:10.3390/md21040204)

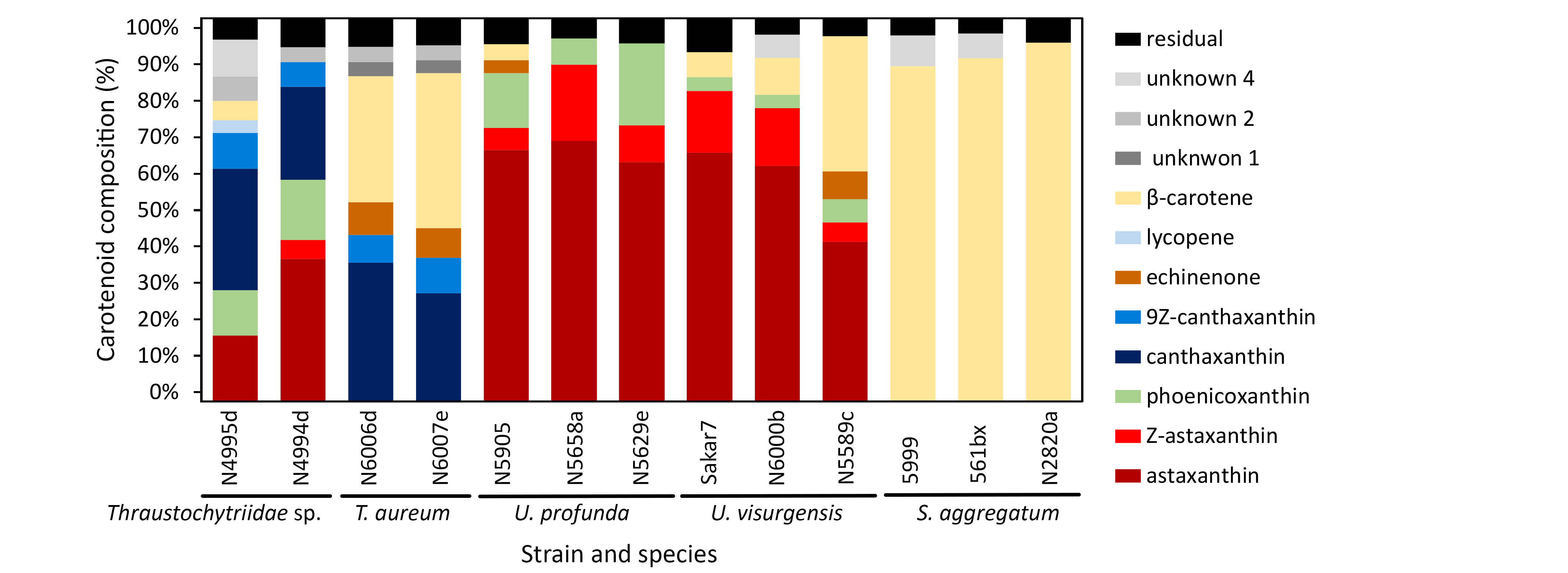

Supplement: Supplementary file 1 [file marinedrugs-21-00204-s001.zip › Figure S1_FW.tif]

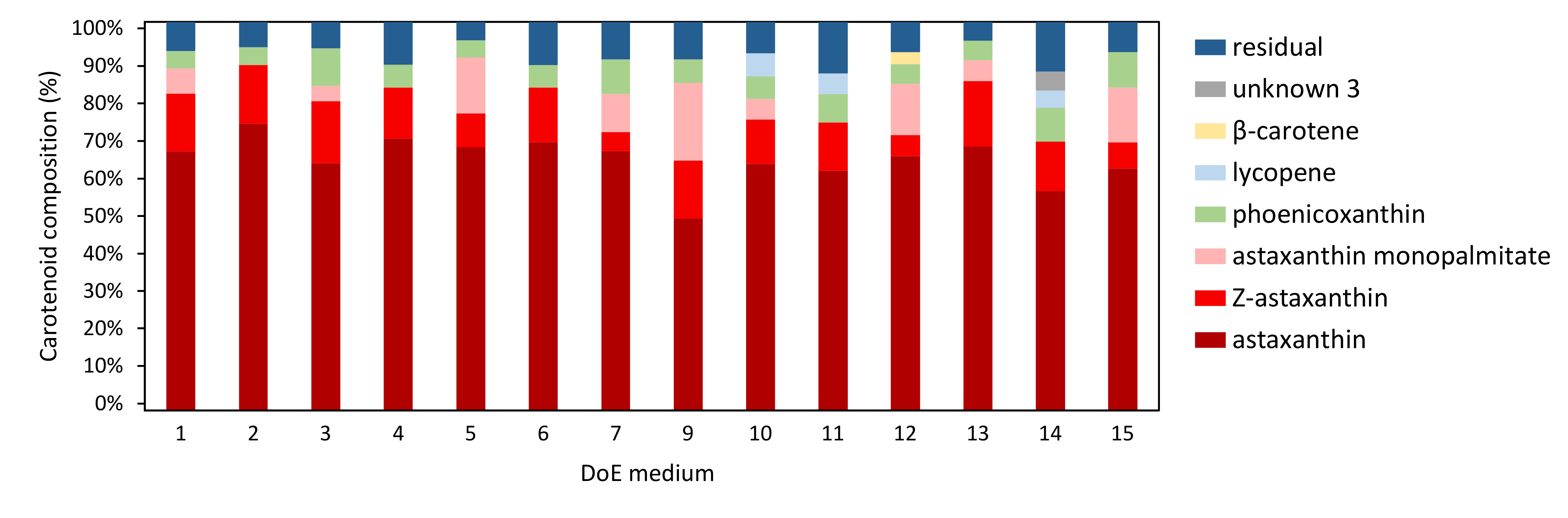

Supplement: Supplementary file 1 [file marinedrugs-21-00204-s001.zip › Figure S2_FW.tif]
